# Single cell RNA sequencing reveals endothelial cell killing and resolution pathways in experimental malaria-associated acute respiratory distress syndrome

**DOI:** 10.1371/journal.ppat.1011929

**Published:** 2024-01-18

**Authors:** Emilie Pollenus, Hendrik Possemiers, Sofie Knoops, Fran Prenen, Leen Vandermosten, Chloë Thienpont, Saeed Abdurahiman, Sofie Demeyer, Jan Cools, Gianluca Matteoli, Jeroen A. J. Vanoirbeek, Greetje Vande Velde, Philippe E. Van den Steen

**Affiliations:** 1 Laboratory of Immunoparasitology, Department of Microbiology, Immunology & Transplantation, Rega Institute for Medical Research, KU Leuven, Leuven, Belgium; 2 Laboratory of Mucosal Immunology, Translational Research in Gastro-Intestinal Disorders (TARGID), Department of Chronic Diseases and Metabolism, KU Leuven, Leuven, Belgium; 3 Laboratory of Molecular Biology of Leukemia, Department of Human Genetics, VIB—KU Leuven, Leuven, Belgium; 4 Centre for Environment and Health, Department of Public Health and Primary Care, KU Leuven, Leuven, Belgium; 5 Biomedical MRI, Department of Imaging & Pathology, KU Leuven, Leuven, Belgium; Deakin University, Australia, AUSTRALIA

## Abstract

*Plasmodium* parasites cause malaria, a global health disease that is responsible for more than 200 million clinical cases and 600 000 deaths each year. Most deaths are caused by various complications, including malaria-associated acute respiratory distress syndrome (MA-ARDS). Despite the very rapid and efficient killing of parasites with antimalarial drugs, 15% of patients with complicated malaria succumb. This stresses the importance of investigating resolution mechanisms that are involved in the recovery from these complications once the parasite is killed. To study the resolution of MA-ARDS, *P*. *berghei* NK65-infected C57BL/6 mice were treated with antimalarial drugs after onset of symptoms, resulting in 80% survival. Micro-computed tomography revealed alterations of the lungs upon infection, with an increase in total and non-aerated lung volume due to edema. Whole body plethysmography confirmed a drastically altered lung ventilation, which was restored during resolution. Single-cell RNA sequencing indicated an increased inflammatory state in the lungs upon infection, which was accompanied by a drastic decrease in endothelial cells, consistent with CD8^+^ T cell-mediated killing. During resolution, anti-inflammatory pathways were upregulated and proliferation of endothelial cells was observed. MultiNicheNet interactome analysis identified important changes in the ligand-receptor interactions during disease resolution that warrant further exploration in order to develop new therapeutic strategies. In conclusion, our study provides insights in pro-resolving pathways that limit inflammation and promote endothelial cell proliferation in experimental MA-ARDS. This information may be useful for the design of adjunctive treatments to enhance resolution after *Plasmodium* parasite killing by antimalarial drugs.

## Author summary

Malaria is a global disease caused by *Plasmodium* parasites, resulting each year in more than 600 000 deaths, mainly caused by complications. Despite rapidly acting antimalarial drugs, 15% of patients who developed a complication still die. Therefore, we aimed to investigate the recovery from a malaria complication, namely malaria-associated acute respiratory distress syndrome (MA-ARDS), after parasite killing with antimalarial drugs. In our mouse model, lung alterations were observed upon infection and lung ventilation was restored during the recovery phase. Moreover, an increased inflammatory state in the lungs was observed upon infection, which was accompanied by a drastic decrease in number of endothelial cells. During resolution, anti-inflammatory pathways were upregulated and remaining endothelial cells multiplied to restore the blood vessel wall. Therefore, promotion of resolution and proliferation of endothelial cells may be an interesting approach to design novel treatments that can be used in combination with the antimalarial drugs.

## Introduction

*Plasmodium* parasites cause malaria, a global disease responsible for more than 200 million clinical cases and 600 000 deaths each year [1]. While most infections remain asymptomatic or result in uncomplicated disease, characterized by fever and fatigue, some infections lead to life-threatening complications. These include cerebral malaria, placental malaria, severe malarial anemia, malaria-associated acute respiratory distress syndrome (MA-ARDS), metabolic and kidney problems [2,3]. MA-ARDS mainly develops in adults and non-immune travelers, with a poor prognosis (mortality up to 80%) [2,3]. This complication is characterized by excessive pulmonary inflammation, resulting in disruption of the alveolar-capillary membrane, which eventually leads to edema, microhemorrhages and hypoxemia [4,5]. A mouse-model resembling the histopathological findings in MA-ARDS patients was developed by infecting C57BL/6 mice with *Plasmodium berghei* NK65 (*Pb*NK65) [6].

The best treatment option currently available for malaria are the artemisinin-based combination therapies. Despite the very rapid and efficient killing of parasites with these therapies, 15% of patients with severe malaria still succumb to the complications [7]. This shows the need to study the recovery, also known as resolution, from these complications, in order to find adjunctive treatments that can be used in combination with the antimalarial drugs.

Resolution of inflammation is an active and coordinated process that aims at the dampening of inflammatory responses and the restoration of tissue function [8–10]. In the early phase of resolution, inflammatory leukocytes go in apoptosis and are removed by phagocytosis by macrophages. This process is called efferocytosis. This induces a change in the macrophage phenotype from an inflammatory to a more pro-resolving phenotype, demonstrating macrophage plasticity. Subsequently, wound healing mechanisms are important to remove debris and restore tissue function. Specialized pro-resolving lipid mediators promote different aspects of the resolution process [11,12].

Previously, we optimized a mouse model to study the resolution of MA-ARDS by treating *Pb*NK65-infected C57BL/6 mice with antimalarial drugs [13]. In this model, a combination of the fast- and short-acting artesunate, which kills the parasite by generating free radicals, is combined with a slower- but longer-acting drug, namely chloroquine [14]. Chloroquine inhibits the heme detoxification process during hemoglobin degradation. The pathogenesis of experimental MA-ARDS has been investigated thoroughly and a pathogenic role for CD8^+^ T cells that attack parasite antigen-presenting endothelial cells was found, thereby causing disruption of the endothelial barrier [4,6,15]. In contrast, much less is known about the leukocytes and mechanisms involved in the recovery from MA-ARDS or severe malaria in general. Therefore, in this study, single cell RNA sequencing was performed on the lungs during the development of MA-ARDS and during the resolution phase after parasite killing, in order to obtain more insights in the dynamics of leukocytes and other pulmonary cells and in the proresolving pathways during the resolution phase. Our data suggest an increased inflammatory state in the lungs during MA-ARDS, which is decreased during the resolution phase. This is accompanied by proliferation of endothelial cells to restore the disrupted endothelial barrier.

## Materials & methods

### Ethics statement

All experiments were performed at the KU Leuven according to the regulations of the European Union (directive 2010/63/EU) and the Belgian Royal Decree of 29 May 2013, and were approved by the Animal Ethics Committee of the KU Leuven (License LA1210186, project P049/2018, P087/2020 and P018/2021, Belgium).

### Mice and experimental design

Seven to eight weeks old C57BL/6 mice were purchased from Janvier Labs (Le Genest-Saint-Isle, France) and housed in individually ventilated cages in a SPF facility. Mice received *ad libitum* water, supplemented with 0.422 mg/ml 4-amino-benzoic acid (PABA; Sigma-Aldrich, Bornem, Belgium) starting at day of infection, and high energy food (Ssniff Spezialdiäte GMBH, Soest, Germany).

Mice were infected with 10^4^ *Pb*NK65-E-infected red blood cells by intraperitoneal (i.p.) injection [6,16]. Sex- and age-matched uninfected controls were included in each experiment. The severity of disease was evaluated daily starting at 6 days post infection (dpi) based on clinical score, body weight and parasitemia, as described previously [13]. Where indicated, mice received antimalarial drugs, which is a combination of artesunate (ART; 10 mg/kg in 0.9% NaCl with 0.1% NaHCO_3_; Sigma-Aldrich) and chloroquine diphosphate salt (CQ; 30 mg/kg in 0.9% NaCl; Sigma-Aldrich), by i.p. injection from 8 dpi until 12 dpi. In case flow cytometry was performed to analyze endothelial cell proliferation, 1.5 mg of bromodeoxyuridine (BrdU) was i.p. injected per mouse at 2 days and 1 day before sacrifice. Mice were euthanized by i.p. injection of 100 μl of dolethal (Vétoquinol, Aartselaar, Belgium; 200 mg/ml). Blood samples were collected by cardiac puncture using heparinized (LEO, Pharma, Lier, Belgium) syringes and broncho-alveolar lavage fluids (BALF) were obtained before transcardial perfusion as described previously [13]. Lungs were collected for further analysis using flow cytometry or single cell RNA sequencing (scRNAseq).

### Whole body plethysmography

Lung ventilation parameters, such as breathing frequency, tidal volume, minute volume, end expiratory pause and enhanced pause ($Penh=\frac{PEP}{PIP}*\frac{Te-Tr}{Tr}$; PEP = peak expiratory pressure, PIP = peak inspiratory pressure, Te = time of expiration, Tr = relaxation time which is the time needed to expire 74% of the tidal volume), were determined at 0 dpi and daily from 6 dpi onwards using a non-restraining Buxco small animal whole body plethysmograph (WBP, Data Sciences International, St. Paul, MN, USA). To prevent measurements of stress, the mice were allowed to acclimatize in the WBP chambers before infection and between 0 and 6 dpi. At a day of measurement, mice were first acclimated for seven minutes (min) before lung ventilation parameters were determined every two seconds for seven min. The data were collected and analyzed using the Buxco FinePointe software (Data Sciences International).

### Micro-Computed Tomography (micro-CT)

A whole-body small animal micro-CT scanner (SkyScan 1278, Bruker micro-CT, Kontich, Belgium) was used to obtain lung micro-CT data of free-breathing anesthesized (isoflurane, 2–2.5% in oxygen, IsoVet, Girovet, Sant Gregori, Italy) animals in supine position. The following scan parameters were used: 50 kVp X-ray source, 1 mm aluminum filter, 350 μA current, 150 ms exposure time per projection and 0.9° increments over a total angle of 220°. Respiration rate and visual information were monitored while scanning. Scanning lasted about 3 minutes in the first and second experiment and 4 minutes in the third experiment. A respiratory weighted reconstructed 3D dataset was obtained with isotopic voxel size of 50 μm. Micro-CT data was reconstructed, visualized and analysed using NRecon, DataViewer and CTan software, provided by the manufacturer. Reconstruction parameters in the NRecon software were: Smoothing = 2 (in the first and second experiment) or 1 (in the third experiment), beam hardening correction = 10%, post-alignment and ring artifact reduction were optimized for each individual scan. In the CTan software, regions of interest, resulting in a volume of interest, were manually delineated on transversal images covering the entire lung, while avoiding the heart and major blood vessels. Subsequently, total lung volume, aerated lung volume and non-aerated lung volume were quantified. A fixed manually set Hounsfield unit (HU) threshold of -465.1 (in first and second experiment) or -401.5 (in third experiment) was used based on the grey density histogram in CTan, with total lung volume ranging from -1040.4 to 645.7 (in first and second experiment) and from -1045.6 to 647.6 (in third experiment) in grey scale indexes, aerated lung volume from -1040.4 to -401.5 (first and second experiment) or from -1045.6 to -401.5 (in third experiment) and non-aerated lung volume from -458.5 to 645.7 (in first and second experiment) and from -394.9 to 647.6 (in third experiment).

### Determination of alveolar edema

The level of alveolar edema was determined by measuring the protein concentration in the BALF supernatant using a Bradford assay (Bio-Rad, CA, USA).

### Lung cell isolation

Lungs were collected in RPMI buffer (RPMI glutamax + 5% FCS + 1% Penicillin/streptomycin) with 0.1% beta-mercaptoethanol at room temperature (RT). Lungs were minced with scissors and incubated in digestion medium containing 2 mg/ml collagenase D (Sigma-Aldrich) and 0.1 mg/ml DNase I (Sigma-Aldrich) for 30 min at 37°C. Afterwards, tissue chunks were minced using needle and syringe and fresh digestion medium was added before a second incubation for 15 min at 37°C. Lung tissue was again minced using a syringe, centrifuged and resuspended using 10 mM EDTA and further diluted in PBS + 2% fetal calf serum (FCS; Gibco, Borgloon, Belgium). RBC lysis was performed with 0.83% ammoniumchloride/10 mM Tris buffer and the cells were passed through a 70 μm nylon cell strainer (VWR, Leuven, Belgium). Live cells were counted in trypan blue in a Bürker chamber.

In case of cell isolation for scRNAseq, debris was removed before counting to further clean up the samples. Here, samples were resuspended in 3.1 ml PBS and transferred to a clean 15 ml tube. 900 μl of cold Debris Removal Solution (MACS Miltenyi Biotec, Leiden, The Netherlands) was added to the cells and mixed well by pipetting. Then 4 ml of cold PBS was added dropwise on top of the cell suspension to obtain two phases. After centrifugation without brake, debris from the top phase and between the two phases was removed. Cells were diluted in cold PBS and centrifuged again. Next, three more washing steps in PBS + 2% FCS were performed before counting in trypan blue in a Bürker chamber.

### Multiplexing individual samples per condition for scRNAseq

After the generation of a single cell suspension and counting of the live cells, 3 x 10^6^ cells per sample were diluted in PBS + 1% bovine serum albumin (BSA, Carl Roth, Karlsruhe, Germany). Aspecific binding of antibodies via Fc receptors was blocked by incubation with Mice Fc block (MACS Miltenyi Biotec) for 10 min at 4°C. Cells were incubated with unique TotalSeq B anti-mouse hashtag antibodies (Biolegend, San Diego, CA, USA) for 30 min at 4°C in order to distinguish between samples. Cells were washed three times in PBS + 1% BSA and four samples per condition, with each a unique hashtag, were pooled together in PBS + 0.04% BSA. Viability and cell counts were assessed using a hematocytometer at the Genomics Core, KU Leuven.

### scRNAseq library preparation and sequencing

Cell suspensions were processed using the 10X Genomics Single Cell 3’ gene expression RNA sequencing kit (10X Genomics, Pleasanton, CA, USA) according to the manufacturer’s instructions. Up to 10 000 live cells were loaded on a 10X Genomics cartridge for each condition. Libraries were sequenced on a NovaSeq6000 (Illumina, San Diego, CA, USA) platform to reach an approximate depth of 25 000 reads per cell. Raw sequencing reads from the gene expression libraries were processed using CellRanger (10X Genomics, version 6.0.2) and reads were mapped to the mm10 reference genome. The 10x single cell library preparation, the sequencing and the CellRanger analysis were performed by the Genomics Core UZ Leuven.

### scRNAseq gene expression analysis

Raw gene expression matrices were merged and analyzed using the Seurat package (version 4.3.0) in Rstudio. Quality control was performed and cells were removed if the number of detected genes was below 200 or above 7000 or when more than 15% of the reads mapped to mitochondrial RNA. Next, normalization of the samples was performed using SCTransform, followed by Harmony to align the different samples. Cells were clustered in Uniform Manifold Approximation and Projection for Dimensionality Reduction (UMAP) plots using the FindClusters function (algorithm = original Louvain algorithm, dimensions = 1:40, resolution = 0.2). The selection of principal components was based on heatmaps and an elbow plot. Differentially expressed genes were determined using the FindAllMarkers function (test = Wilcoxon Rank Sum test, log fold change (logFC) threshold = 0.25, only positive = TRUE). Cell clusters were identified based on the differentially expressed genes (obtained from both the FindAllMarkers and FindConservedMarkers function), established marker genes available in the literature and SingleR with ImmGen and Mouse.RNAseq as reference databases. The relative frequency of each cluster was calculated in each condition or in each sample. Relative frequencies per sample and the absolute cell count obtained during cell isolation using the Bürker chamber were used to calculate the absolute number of each population. We next isolated each major subset (lymphoid, myeloid and non-immune) and repeated the analysis. For the lymphoid cell analysis, following clusters were extracted from the original analysis: B cells, γδT cells/innate lymphoid cells (gdT/ILC), NK cells, effector T cells (Teff), naïve T cells (Tnaive) and dividing or expanding T cells (Expanding_T). Clustering was again performed with the FindClusters function (algorithm = original Louvain algorithm, dimensions = 1:25, resolution = 0.5). For the myeloid cell analysis, following clusters were extracted: neutrophils (Neutros), dendritic cells (DC), monocytes/macrophages (Monos/Macros), alveolar macrophages (AM) and clustering was performed using FindClusters (algorithm = original Louvain algorithm, dimensions = 1:23, resolution = 0.5). In case of the non-immune cell analysis, extracted clusters were: endothelial cells (ECs), aerocytes (Aeros), type 1 alveolar epithelial cells (Epc_AT1), type 2 alveolar epithelial cells (Epc_AT2), fibroblasts (Fibro), mesothelial cells (Meso), lymphatic ECs (LECs). FindClusters (algorithm = original Louvain algorithm, dimensions = 1:16, resolution = 0.2) was used for clustering. Cell type information of the different subcluster analyses was incorporated into the original Seurat object for further downstream analyses.

### Pseudobulk analysis and gene set enrichment analysis (GSEA) between conditions

To further investigate changes between conditions within a cluster, pseudobulk analysis was performed. Aggregated expression values were calculated within each cluster using AggregateExpression. Counts from the cluster of interest were extracted and DESeq2 was performed to identify differentially expressed genes. LfcShrink using the apelgm method was used to correct for differences in baseline expression level [17]. Log2FoldChange of all differentially expressed genes was used to perform GSEA analysis using the fgsea package (version 1.26.0) and the MSigDB Hallmark gene sets, which were acquired from the Molecular Signatures Database using the msigdbr function. Significantly enriched gene sets were selected based on an adjusted p value of < 0.05 and the normalized enrichment score (NES) was visualized to determine whether these gene sets were up- or downregulated in a given condition.

### Cell-cell communication

The MultiNicheNet package (version 1.0.3) was used to determine interactions between cell clusters based on information about ligands, receptors and ligands within signaling pathways. Each condition was compared to the two other conditions to determine specific cell-cell communication in the condition of interest. The default MultiNicheNet pipeline was used. Some subclusters were combined to obtain larger clusters per sample and therefore more robust results and avoid drop-outs of clusters due to low numbers. Specifically, arterial blood ECs (BECs_Art), stressed arterial BECs (BECs_Art_Stressed), venous BECs (BECs_Ven), proliferating BECs (BECs_Proliferating) and Aerocytes are clustered together into the cluster BECs. CD103^+^ (CD103^+^ DCs) and CD11b^+^ dendritic cells (CD11b^+^ DCs) are combined into a DCs cluster. Type 1 alveolar (EpC_AT1), type 2 alveolar (EpC_AT2) and ciliated epithelial cells (EpC_Ciliated) are combined into an EpCs cluster. Col13a1^+^ (Fibro_Col13a1) and Col14a1^+^ fibroblasts (Fibro_Col14a1) are clustered together as Fibro. The three macrophage clusters (Macros, Macros_2 and Macros_3) are combined into one Macros cluster. The effector T cell cluster (Teff) comprises the three effector T cell subclusters (Teff, Teff_2, Teff_cytotox), the type I interferon-responding T cells (IFN-resp. T cells) and the expanding T cells (Expanding_T). Two neutrophil clusters (Neutros & Neutros_2) are combined into one Neutros cluster and the γδ T cells and ILC2 are combined into a gdT-ILC cluster.

Clusters were only used in the calculation if more than 10 cells were present in at least 2 samples per condition. First, cell-cell communication between all clusters was determined and the top 30 ligands per condition were selected for visualization in a circos plot. Next, analysis was repeated with effector T cells (Teff) as sender and blood endothelial cells (BECs) as receiver or with Teff as receiver and BECs as sender. Furthermore, analysis was performed with all clusters as senders and BECs as receiver. In addition, the ligand-receptor expression per sample, the predicted ligand-target links and the expression of the predicted target genes were visualized across samples with BECs as receiver following the default pipeline.

### Flow cytometry on pulmonary endothelial cells

3 million cells per sample were washed with PBS. Cells were stained with Zombie Aqua (1/1000; Biolegend) in combination with Mice Fc block (MACS Miltenyi Biotec), for 15 min at RT in the dark. After washing twice with PBS + 2% FCS + 2 mM EDTA, cells were incubated with a mixture of monoclonal antibodies (S1 Table) dissolved in PBS with Brilliant stain buffer (BD Biosciences; Erembodegem, Belgium) for 20 min at 4°C in the dark. To stain for the injected BrdU, intranuclear staining was performed following the surface staining as described above. Cells were fixed and permeabilized after two wash steps with PBS, using Cytofix/Cytoperm buffer (BD Biosciences) for 20 min at RT in the dark. After washing with Perm/Wash buffer (BD Biosciences), cells were further fixed and permeabilized using the Cytoperm Permeabilization Buffer Plus (BD Biosciences) for 10 min at 4°C in the dark. Cells were washed again using Perm/Wash buffer and a last fixation and permeabilization step was performed for 5 min at RT in the dark using the Cytofix/Cytoperm buffer. After washing with Perm/Wash buffer cells were treated with 30 μg of DNase I for 1h at 37°C in the dark to expose incorporated BrdU. Cells were washed with Perm/Wash buffer and incubated for 20 min in the dark at RT with anti-BrdU antibodies followed by two wash steps with Perm/Wash buffer.

Per sample, 200 000 live single cells were analyzed using the BD Fortessa X-20 Flow cytometer (BD Biosciences). Data analysis was performed in the FlowJo v10 software (FlowJo LLC, Ashland, OR, USA). Endothelial cells were identified as CD45^-^ CD31^+^ (gating strategy in S1 Fig) and the absolute number was calculated by multiplying the frequency of ECs within total live cells by the number of live cells counted in the Bürker chamber. The frequency of ECs expressing a certain marker and the mean fluorescent intensity of that marker on the ECs was determined.

### Statistical analysis

The GraphPad PRISM software (GraphPad, San Diego, California, USA) was used for statistical analysis. The non-parametric Mann-Whitney U test followed by the Holm-Bonferroni correction was used. Significance was determined between all groups and p-values were indicated as follows: *p<0.05, **p<0.01, ***p<0.001. In the time course graphs, mean and standard error of the mean were shown. In the column graphs, each symbol represented a different mouse and the median in each group was indicated by a horizontal black line. Statistical differences compared to the uninfected controls are indicated with asterisk above/below the data sets and horizontal lines with asterisk on top indicate significant differences between infected groups.

## Results

### Experimental MA-ARDS is characterized by pulmonary edema and altered lung ventilation

**Fig 1:**
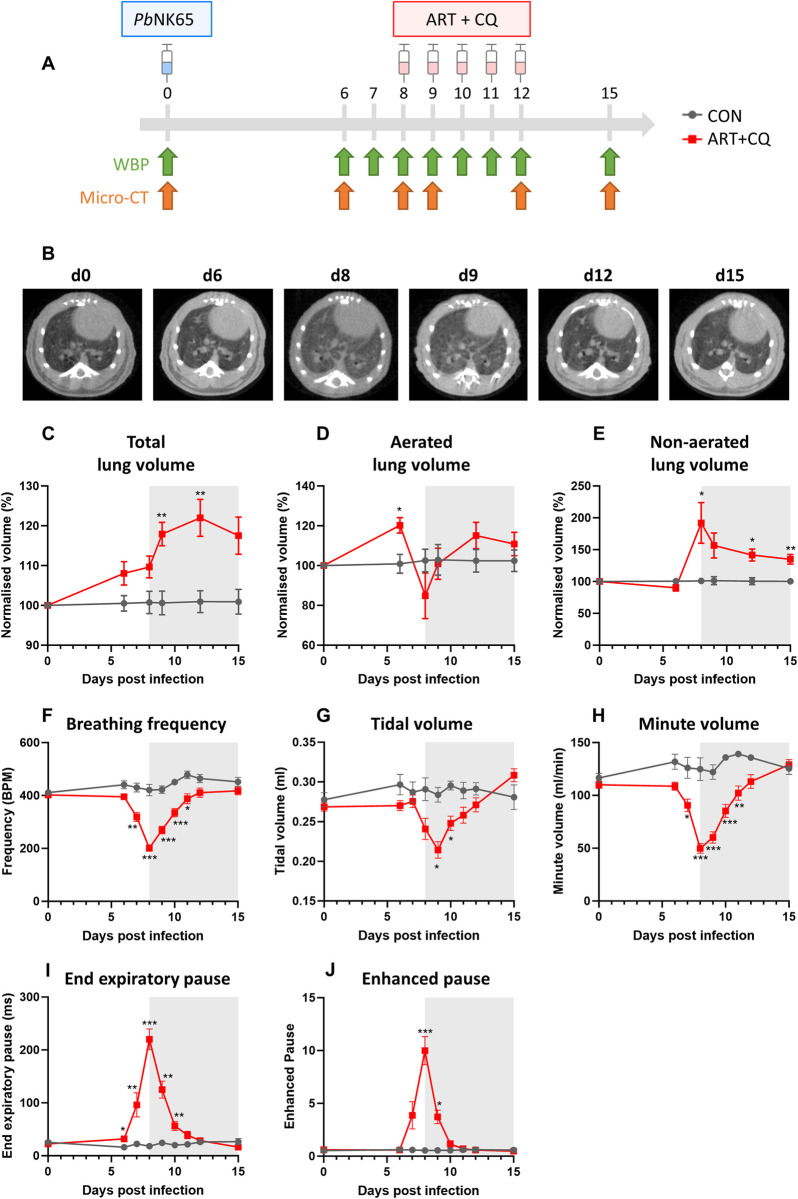
Alterations in the lung during MA-ARDS pathology and resolution. *Pb*NK65-infected C57BL/6 mice were treated daily from 8 until 12 dpi with 10 mg/kg artesunate + 30 mg/kg chloroquine (ART+CQ). (A) Schematic representation of the mouse model and time-points of whole body plethysmography (WBP) and Micro-Computed Tomography (Micro-CT) measurements. (B) Representative images of Micro-CT scans are shown. (C-E) Micro-CT was performed at 0, 6, 8, 9, 12 and 15 dpi and total lung volume (C), aerated lung volume (D) and non-aerated volume (E) were calculated. Volumes were normalized to the uninfected controls and to 0 dpi. Data from three experiments. Data are represented as means ± SEM. n = 8–10 for uninfected controls (CON), n = 9–18 for ART+CQ-treated, *Pb*NK65-infected C57BL/6 mice (ART+CQ). (F-J) Whole body plethysmography was performed at 0, 6, 7, 8, 9, 10, 11, 12 and 15 dpi and lung ventilation parameters, such as breathing frequency (F), tidal volume (G), minute volume (H), end expiratory pause (I) and enhanced pause (J) were calculated. Data from two experiments. Data are represented as means ± SEM. n = 6 for CON, n = 13–27 for ART+CQ.

Infection of C57BL/6 mice with *Pb*NK65 results in the development of MA-ARDS with clinical symptoms appearing at 8 dpi (Figs 1A and S2) [13]. Therefore, antimalarial treatment with a combination of artesunate and chloroquine (ART+CQ) is given daily from this day until 12 dpi. In our previous study, we described that at 12 dpi, alveolar edema was partially but not yet completely resolved and that infiltrating leukocyte numbers in the lung further increased [13]. Therefore, we previously concluded that at 12 dpi resolution of inflammation and tissue damage was actively happening, while the resolution phase was largely finished by 15 dpi. Similarly, to what we described previously, parasitemia already decreased after one treatment and all parasites were cleared by 12 dpi (S2A Fig). Clinical score and body weight still worsened at 9 dpi, but from 10 dpi onwards mice started to recover (S2B and S2C Fig).

Here, we further characterized changes in lung phenotype and ventilation at different time-points during the development and resolution of MA-ARDS (Fig 1A). Micro-CT scans of the lung region were generated at the different time-points (Fig 1A and 1B). A general hyperdensification in the lung area was observed at 8 and 9 dpi, suggesting the presence of pulmonary edema and inflammation. A gradual increase in total lung volume, which was a trend at 6 and 8 dpi (p = 0.0557 and p = 0.0592, respectively), but became significant from 9 dpi onwards, was observed, potentially reflecting a compensatory enlargement of the lung to compensate for loss of airspaces due to edema (Fig 1C). This was accompanied by an increase in aerated lung volume at 6 dpi when edema did not develop yet (Fig 1D). In contrast, from 8 dpi onwards, no increased aerated lung volume was observed anymore. This was accompanied by a striking increase in non-aerated lung volume at 8 dpi, suggesting the development of lung edema (Fig 1E). During resolution, aerated lung volume was restored and a partial decrease in non-aerated volume was observed. We also examined lung ventilation parameters using whole body plethysmography. Upon infection, a decrease in breathing frequency (Fig 1F) and tidal volume (Fig 1G) was observed and both parameters were restored during the resolution phase, indicating that resolution of inflammation and tissue damage indeed rescues lung ventilation. Minute volume, which combines breathing frequency and tidal volume and is therefore an indicator for the amount of gas exchange that can occur, was significantly decreased upon infection and also restored during resolution (Fig 1H). A massive increase in end expiratory pause (Fig 1I) and enhanced pause (Fig 1J) was also observed upon infection, suggesting a delay in the transition from expiration to the next inspiration. These increased pauses contribute to the decreased breathing frequency, and also quickly return to baseline during the resolution phase.

### Dynamics of pulmonary cell subsets during the development and resolution of MA-ARDS

**Fig 2:**
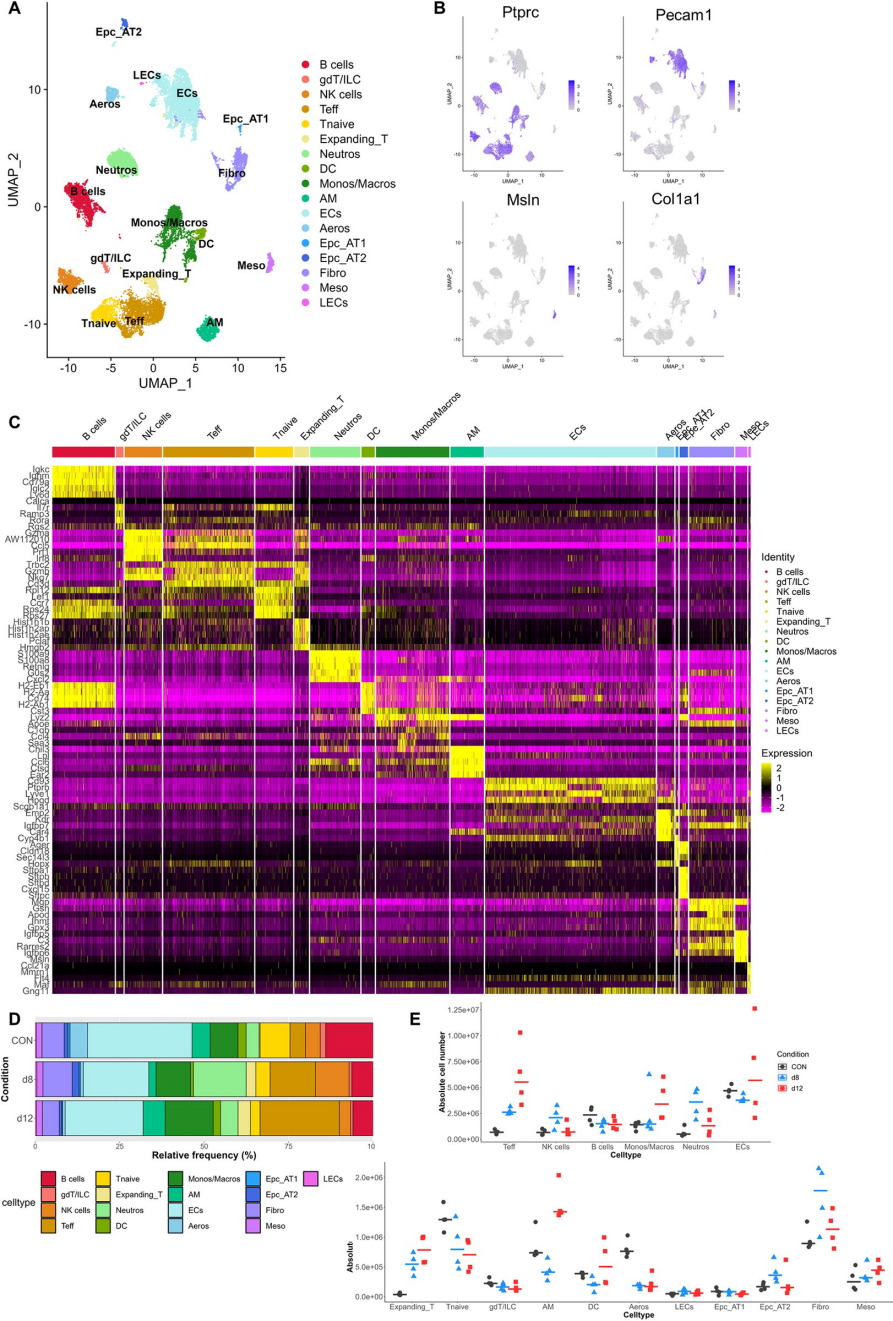
Dynamics in cellular composition of the lung during development and resolution of MA-ARDS. *Pb*NK65-infected C57BL/6 mice were treated daily from 8 until 12 dpi with 10 mg/kg artesunate + 30 mg/kg chloroquine (ART+CQ). Mice were dissected at 8 dpi (when symptoms of MA-ARDS are present and before start of antimalarial treatment; d8) or at 12 dpi (when mice are recovering from MA-ARDS after parasite killing; d12). Uninfected mice were used as controls (CON). Cells were isolated from the lungs and single cell RNA sequencing (scRNAseq) was performed. (A) UMAP plot of all scRNAseq data showing 17 distinct cell types. (B) UMAP plots visualizing expression levels of canonical markers of leukocytes (*Ptprc*), endothelial cells (*Pecam1*), mesothelial cells (*Msln*) and fibroblasts (*Col1a1*). (C) Heatmap displaying the top 5 markers of each cell type and its expression across all individual cells clustered per cell type. (D) Frequency plot showing the relative abundance of each cell type per condition within the lymphoid cells. (E) Absolute number of each cell type per sample was calculated. Each symbol represents an individual mouse. Horizontal lines represent the median. (A-E) n = 4 for CON, d8 and d12 (4 mice per condition were pooled using Hashtag oligos). gdT/ILC, γδ T cells/innate lymphoid cells; NK, natural killer; Teff, effector T cells; Tnaive, naïve T cells; Expanding_T, dividing or expanding T cells; Neutros, neutrophils; DC, dendritic cells; Monos/Macros, monocytes/macrophages; AM, alveolar macrophages; ECs, endothelial cells; Aeros, aerocytes; EpC_AT1, type 1 alveolar epithelial cells; EpC_AT2, type 2 alveolar epithelial cells; Fibro, fibroblasts; Meso, mesothelial cells; LECs, lymphatic endothelial cells.

In order to obtain a detailed characterization of the dynamics of cellular transcriptomes in the lung during the development and resolution of MA-ARDS, scRNAseq analysis was performed on pulmonary cells obtained from the lungs of uninfected control mice (CON), untreated, *Pb*NK65-infected mice with MA-ARDS at 8 dpi (d8) and ART+CQ-treated mice recovering from MA-ARDS at 12 dpi (d12). Four mice were selected per condition based on the level of alveolar edema present and pulmonary single cell suspension was used for scRNAseq analysis (S3 Fig). In total, 16.085 single cells passed quality control and were clustered based on their expression profile using the Seurat package (Fig 2A and S2 Table) [18]. In total, 17 clusters were identified, including lymphoid and myeloid cells, endothelial cells, epithelial cells, fibroblasts and mesothelial cells (Fig 2B and 2C and S2 Table). Interestingly, dynamic changes in multiple populations were found (Fig 2D and 2E). Both in frequency (Fig 2D) and absolute number (Fig 2E), effector T cells (Teff) were found to increase upon infection, while the naïve T cells (Tnaive) decreased. The number of neutrophils also increased upon infection, while the number of monocytes and macrophages increased only by d12. Alveolar macrophages (AM) and dendritic cells decreased in the lung upon infection and returned to baseline during resolution. Interestingly, a decrease in aerocytes was observed at d8 and their number remained low at d12. Aerocytes are described as the specialized capillary endothelial cells that align the alveolar epithelial cells and are therefore mainly involved in gas exchange and leukocyte trafficking [19,20]. In the subsequent sections, a more detailed analysis of specific cell subsets was performed by subclustering analysis of lymphoid, myeloid and non-immune cell populations separately.

### Pulmonary lymphocytes during the development and resolution of MA-ARDS

**Fig 3:**
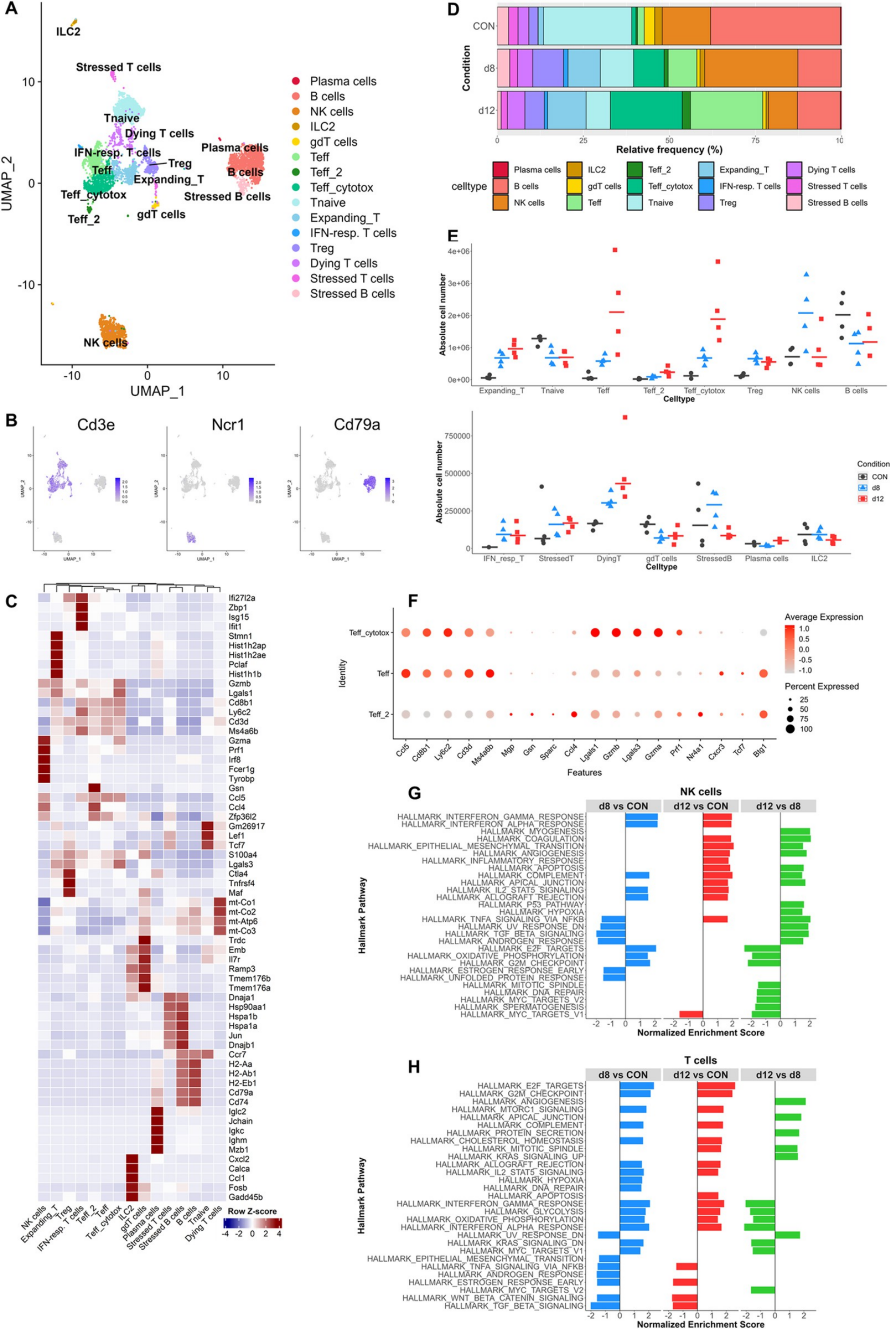
Dynamics of lymphocyte populations in the lung during development and resolution of MA-ARDS. Lymphoid cell clusters (B cells, gdT/ILC, NK cells, Teff, Tnaive and Expanding_T) from the original lung scRNAseq analysis of control (CON), *Pb*NK65-infected (d8) and ART+CQ-treated (d12) mice (Fig 2) were extracted and subclustering was performed. (A) UMAP plot of lymphoid cell data showing 15 distinct cell types. (B) UMAP plots visualizing expression levels of canonical markers of T cells (*Cd3e*), NK cells (*Ncr1*) and B cells (*Cd79a*). (C) Heatmap displaying the top 5 markers of each cell type and its expression across all cell types. (D) Frequency plot showing the relative abundance of each cell type per condition within the total scRNAseq data. (E) Absolute number of each cell type per sample was calculated. Each symbol represents an individual mouse. Horizontal lines represent the median. (F) Dotplots showing the average and percent expression of effector T cell genes in the Teff, Teff_2 and Teff_cytotox cluster. (G) Gene set enrichment analysis was performed on the differentially expressed genes obtained through pseudobulk analysis of NK cells and significant Hallmark pathways are shown. Positive/negative normalized enrichment scores represent an upregulation/downregulation of the pathway in the condition of interest (first in comparison) versus control condition (second in comparison). (H) Gene set enrichment analysis was performed on the differentially expressed genes obtained through pseudobulk analysis of all T cells combined (Tnaive, Teff, Teff_2, Teff_cytotox, Treg, Expanding_T, IFN-resp. T cells) and significant Hallmark pathways are shown. Positive normalized enrichment scores represent an upregulation of the pathway in the condition of interest (first in comparison) versus control condition (second in comparison). (A-H) n = 4 for CON, d8 and d12 (4 mice per condition were pooled using Hashtag oligos). NK, natural killer; ILC2, innate lymphoid cells type 2; Teff, effector T cells; Teff_cytotox, cytotoxic effector T cells; Tnaive, naïve T cells; Expanding_T; dividing or expanding T cells; IFN-resp., type I interferon-responding; Treg, regulatory T cells.

To further analyze the impact of MA-ARDS and its resolution, lymphoid cells, comprising of T cells, B cells, NK cells and innate lymphoid cells (ILCs), were subclustered and analyzed in more detail. This revealed 10 different T cell clusters, one cluster of type 2 ILCs, one cluster of NK cells and three clusters of B cells, including one small cluster of plasma cells, based on their expression profile (Fig 3A–3C and S3 Table). CD8^+^ T cells are pathogenic in the development of MA-ARDS by killing parasite antigen-presenting ECs [4,6,15], and indeed an expansion in Teff and cytotoxic Teff cells was observed in the lungs at d8 (Fig 3D and 3E). Their number further increased during resolution. A population of type I IFN-responding T cells was also found at d8 and d12, while this population was almost absent in control mice. In addition, the number of dividing or expanding T cells and regulatory T cells were also increased after infection. In contrast, the number of naïve T cells and γδ T cells decreased upon infection. NK cells also expanded upon infection, while B cells seemed to decrease upon infection. Stressed B and T cells were identified based on marker genes related to the stress response, such as heat shock proteins, and these populations increased in number upon infection. Three different Teff populations were found (Fig 3A). Therefore, the markers distinguishing these populations were investigated in more detail (Fig 3C and 3F). One cluster (Teff_cytotox) clearly expressed the highest levels of cytotoxic markers, such as granzyme A, granzyme B (*GzmB*) and perforin across all conditions (Figs 3C, 3F, and S4A). *GzmB* appeared to be upregulated at d8 compared to CON in different lymphoid cell populations, such as plasma cells, NK cells, all T effector populations, expanding T cells and IFN-responding T cells (S4A Fig). Expression of *GzmB* was still high on most populations at 12 dpi. A small cluster of Teff cells (Teff_2) was found to express markers, such as *Mgp*, *Gsn*, *Sparc* and *Zfp36l2*, suggesting that this population is undergoing clonal expansion (Fig 3C and 3F). While *Ccl5* was produced by all effector T cell populations, *Ccl4* expression appeared specific for the Teff_2 population (Fig 3C–3F). Other T cell populations and NK cells were also found to express both *Ccl4* and *Ccl5* upon infection (S4B and S4C Fig). *Ccl4* and *Ccl5* play a role in the recruitment of pro-inflammatory cells to the site of inflammation.[21] In general, both *Ccl4* and *Ccl5* expression was increased on most populations after infection and even further during resolution (S4B and S4C Fig).

GSEA analysis revealed that NK cells are activated upon infection as an increase in interferon-γ (IFN-γ) response, interferon-α (IFN-α) response and complement were found (Fig 3G and S4 Table). Moreover, this population was found to be proliferating, which correspond with an increased number at 8 dpi. During resolution, anti-inflammatory and pro-resolving pathways, such as transforming growth factor β (TGF-β) signaling, apoptosis and angiogenesis, seemed to be restored or upregulated in NK cells, while pathways related to cell cycle and proliferation were decreased.

In concordance with NK cells, increased proliferation was observed at d8 in the global T cell population (Fig 3H and S5 Table). In addition, different pro-inflammatory pathways are upregulated at d8, which corresponds with T cells being a crucial mediator in the immunopathology of MA-ARDS. At d12, some of these inflammatory pathways were down compared to d8, but still up compared to CON. These results confirm the highly inflammatory state of the lungs upon infection, but homeostasis is not yet fully achieved at d12.

### Dynamics of myeloid cells in the lung during MA-ARDS and its resolution

**Fig 4:**
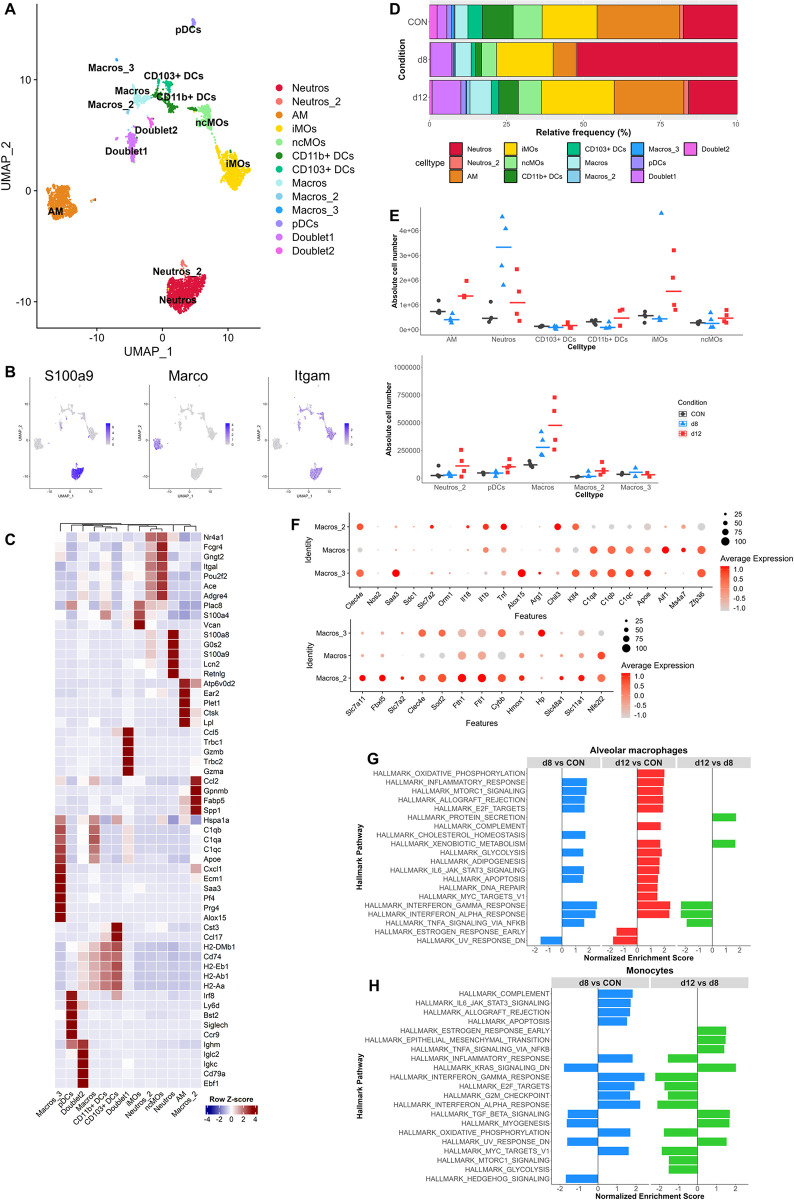
Changes in pulmonary myeloid cell populations in MA-ARDS and its resolution. Myeloid cell clusters (Neutros, DC, Monos/Macros, AM) from the original lung scRNAseq analysis of control (CON), *Pb*NK65-infected (d8) and ART+CQ-treated (d12) mice (Fig 2) were extracted and subclustering was performed. (A) UMAP plot of myeloid cell data showing 13 distinct cell types. (B) UMAP plots visualizing expression levels of canonical markers of neutrophils (*S100a9*), alveolar macrophages (*Marco*) and general myeloid cells (*Itgam*). (C) Heatmap displaying the top 5 markers of each cell type and its expression across all cell types. (D) Frequency plot showing the relative abundance of each cell type per condition within the myeloid cells. (E) Absolute number of each cell type per sample was calculated. Each symbol represents an individual mouse. Horizontal lines represent the median. (F) Dotplots showing the average and percent expression of M1-M2 marker genes (upper panel) and genes related to heme and iron metabolism (lower panel) in the Macros, Macros_2 and Macros_3 cluster. (G) Gene set enrichment analysis was performed on the differentially expressed genes obtained through pseudobulk analysis of alveolar macrophages and significant Hallmark pathways are shown. Positive/negative normalized enrichment scores represent an upregulation/downregulation of the pathway in the condition of interest (first in comparison) versus control condition (second in comparison). (H) Gene set enrichment was performed on the differentially expressed genes obtained through pseudobulk analysis of all monocytes combined (iMOs, ncMOs) and significant Hallmark pathways are shown. Positive normalized enrichment scores represent an upregulation of the pathway in the condition of interest (first in comparison) versus control condition (second in comparison). (A-H) n = 4 for CON, d8 and d12 (4 mice per condition were pooled using Hashtag oligos). Neutros, neutrophils; AM, alveolar macrophages; iMOs, inflammatory monocytes; ncMOs, non-classical monocytes; DCs, dendritic cells; Macros, macrophages; Monos, monocytes; pDCs, plasmacytoid dendritic cells.

The myeloid cell populations, including neutrophils, monocytes/macrophages, dendritic cells and AM, after re-clustering resulted in 13 different clusters (Fig 4A–4C and S6 Table). Two clusters, named Doublet1 and Doublet2, expressed myeloid markers together with typical T and B cell markers, respectively (Fig 4C). Addition of these clusters to the lymphoid cell analysis, resulted again in separate clusters with monocyte and macrophage markers as marker genes (S5 Fig). These findings suggest that these populations are probably doublets that either escaped prior filtering or that these are physiologically relevant doublets of monocytes adhering to T cells and B cells, respectively. An increase in numbers of neutrophils and macrophages was observed upon infection, while the AM, CD103^+^ and CD11b^+^ dendritic cells and non-classical monocytes (ncMOs) decreased (Fig 4D and 4E). During the resolution phase, AM, CD103^+^ and CD11b^+^ dendritic cells and ncMOs increased again in the lungs, and the number of macrophages and inflammatory monocytes (iMOs) further increased. A decrease in neutrophils compared to d8 was observed.

In addition to the main macrophage cluster (Macros), two small extra clusters of macrophages (Macros_2 and Macros_3) were found (Fig 4A). All three macrophage populations appeared to express both M1 (*Clec4e*, *Saa3*, *Il1b*, *Tnf*) and M2 marker genes (*Arg1*, *Chil3*, *Klf4*, *C1qa-c*, *Apoe*, *Aif1*, *Ms4a7*, *Zfp36*. M2 markers related to complement and clearance of apoptotic cells were expressed by Macros and Macros_3 (Fig 4C and 4F, upper panel). Macros_3 population was found to express 15-lipoxygenase (*Alox15*), an enzyme involved in the production of specialized pro-resolving lipid mediators, especially during the resolution phase (Fig 4C and 4F, upper panel; S6A Fig) [22,23]. *Alox15* expression was found to be potentiated by efferocytosis in human M2 macrophages [22]. In addition, Macros_3 also expressed proteoglycan 4 (*Prg4)*, which is known to inhibit the recruitment of M1 macrophages and apolipoprotein E (*Apoe)*, which was found to suppress T cell activation and proliferation (Fig 4C and 4F, upper panel) [24–26]. On the contrary, also pro-inflammatory genes, such as platelet factor 4 (*Pf4)*, *Cxcl1* and serum amyloid A3 (*Saa3)* were found in the Macros_3 population. The Macros_2 population showed increased expression of genes related to hemoglobin degradation (*Hp*, *Slc48a1*, *Slc11a1*, *Hmox1*) and heme-iron metabolism (*Nfe2l2*, *Fth1*, *Ftl1*, *Sod2*, *Slc7a11*, *Fbxl5*, *Slc7a2*), indicative of an iron excess in these macrophages (Fig 4F, lower panel, S6B and S6C Fig) [27]. The highest expression of *Hmox1* expression was found at d8 in this Macros_2 population, while *Fth1* expression was found to be increased at d8 in different myeloid cell populations and appeared to be even higher at d12 in some populations, such as the Macros_2 (S6B and S6C Fig). These observations, in combination with the increased number of Macros_2 during resolution (Fig 4E), suggest that these cells are phagocytosing (infected) red blood cells and debris after parasite killing.

GSEA analysis revealed the self-renewing capacity (mtorc1 signaling, E2F targets and DNA repair) of the remaining AM in an attempt to replace the AM that underwent apoptosis upon infection (Fig 4G and S7 Table). Also an increased pro-inflammatory state was observed upon infection, while this was decreased during resolution. In the monocytes (iMOs and ncMOs combined), increased inflammation and increased proliferation was also observed at d8, and these were both decreased during resolution (Fig 4H and S8 Table). No significant pathways were obtained for the monocytes when comparing d12 to CON, suggesting that the expression profile of the monocytes at d12 has returned back to baseline. A mixed inflammatory state (increased tumor necrosis factor-α (TNF-α) signaling and restored TGF-β signaling) was observed during resolution.

### Changes in non-immune cells in the lungs during the development and resolution of MA-ARDS

**Fig 5:**
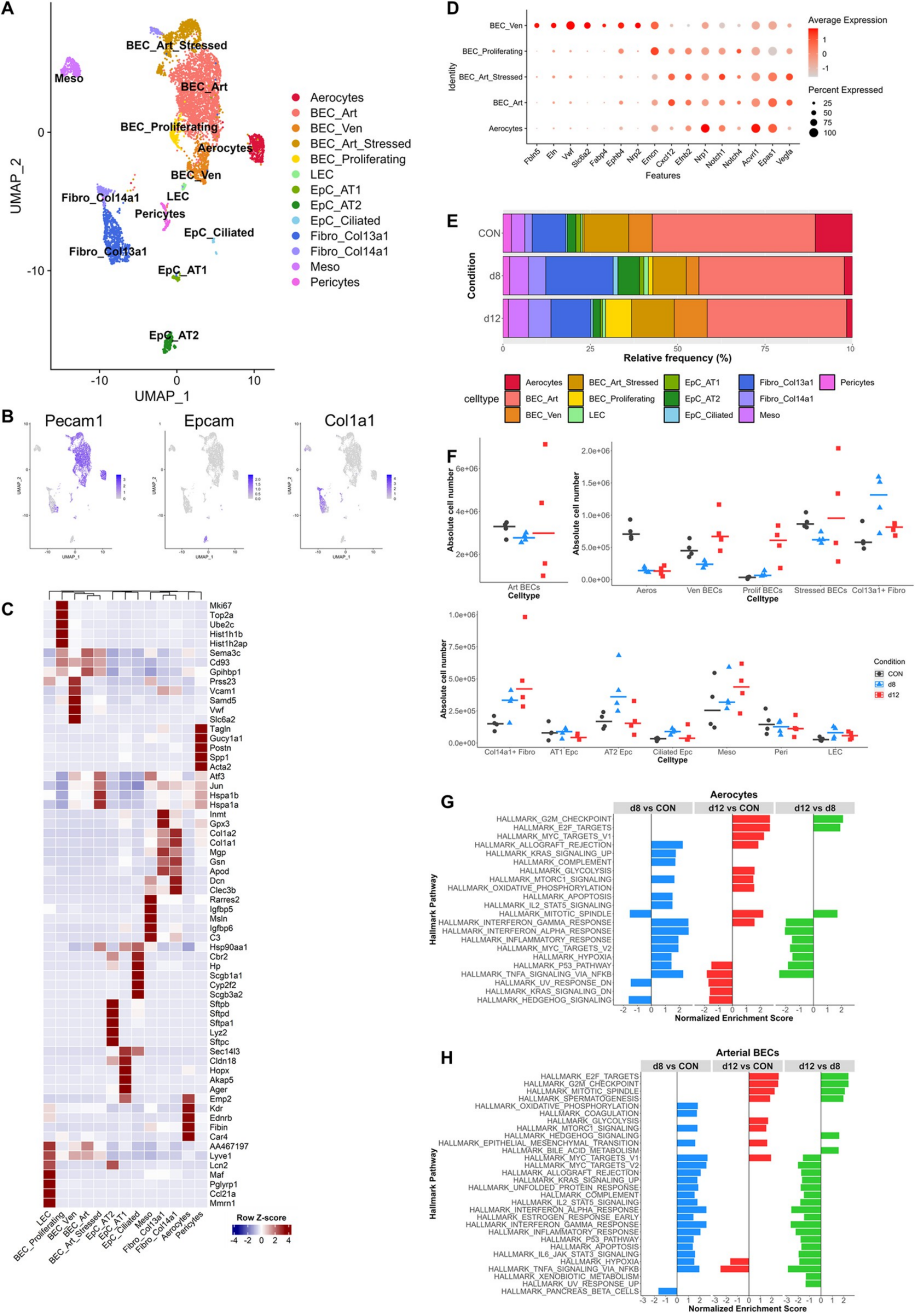
Changes in pulmonary nonimmune cell populations in MA-ARDS and its resolution. Nonimmune cell clusters (ECs, Aeros, Epc_AT1, Epc_AT2, Fibro, Meso, LECs) from the original lung scRNAseq analysis of control (CON), *Pb*NK65-infected (d8) and ART+CQ-treated (d12) mice (Fig 2) were extracted and subclustering was performed. (A) UMAP plot of nonimmune cell data showing 13 distinct cell types. (B) UMAP plots visualizing expression levels of canonical markers of endothelial cells (*Pecam1*), epithelial cells (*Epcam*) and fibroblasts (*Col1a1*). (C) Heatmap displaying the top 5 markers of each cell type and its expression across all cell types. (D) Dotplot showing the average and percent expression of genes distinguishing between arterial and venous endothelial cells, according to Hurskainen *et al*.[20] and dela Paz *et al*.[28] in the BECs (BEC_Art, BEC_Art_Stressed, BEC_Ven, BEC_proliferating, Aerocytes). (E) Frequency plot showing the relative abundance of each cell type per condition within the nonimmune cells. (F) Absolute number of each cell type per sample was calculated. Each symbol represents an individual mouse. Horizontal lines represent the median. (G) Gene set enrichment analysis was performed on the differentially expressed genes obtained through pseudobulk analysis of aerocytes and significant Hallmark pathways are shown. Positive/negative normalized enrichment scores represent an upregulation/downregulation of the pathway in the condition of interest (first in comparison) versus control condition (second in comparison). (H) Gene set enrichment was performed on the differentially expressed genes obtained through pseudobulk analysis of arterial BECs and significant Hallmark pathways are shown. Positive normalized enrichment scores represent an upregulation of the pathway in the condition of interest (first in comparison) versus control condition (second in comparison). (A-H) n = 4 for CON, d8 and d12 (4 mice per condition were pooled using Hashtag oligos). BEC_Art, arterial blood endothelial cells; BEC_Ven, venous blood endothelial cells; BEC_Proliferating, proliferating blood endothelial cells; LEC, lymphatic endothelial cells; EpC_AT1, type 1 alveolar epithelial cells; EpC_AT2, type 2 alveolar epithelial cells; EpC_Ciliated, ciliated epithelial cells; Fibro_Col13a1, Col13a1^+^ fibroblasts; Fibro_Col14a1, Col14a1^+^ fibroblasts; Meso, mesothelial cells.

Also, all non-immune cells were selected and clustered again. This revealed different clusters of blood endothelial cells (BECs) and one cluster of lymphatic ECs, three epithelial cell clusters and multiple clusters of fibroblasts, mesothelial cells and pericytes (Fig 5A and 5B). Aerocytes, clustered separately from the other BECs, indicating a unique expression pattern (Fig 5A). Marker genes for this population are *Emp2*, *Kdr*, *Ednrb*, *Fibin* and *Car4* (Fig 5C and S9 Table) [20]. In concordance with Hurskainen *et al*. and dela Paz *et al*., a BEC population expressing markers such as *Fbln5*, *Eln*, *Vwf*, *Slc6a2*, *Fabp4*, *Ephb4*, *Nrp2* and *Emcn*, was identified as venous BECs while the largest BEC population expressed *Cxcl12*, *Efnb2*, *Nrp1*, *Notch1*, *Notch4*, *Acvrl1*, *Epas1* and *Vegfa* and are thus identified as arterial BECs (Fig 5D) [20,28]. Vascular endothelial growth factor α (*Vegfa*) plays an important role in vascular development and proliferation of endothelial cells [29]. *Vegfa* was also expressed by other cell types, such as epithelial cells, fibroblasts and pericytes (S7A Fig). Levels of *Vegfa* mRNA were decreased upon infection and restored during resolution in the nonimmune cells. However, it is known that mRNA levels of VEGF diverge from protein levels in this model [15].

Upon infection (d8), a decrease in BECs, mainly in aerocytes and venous BECs, was observed (Fig 5F). This may be responsible for the edema development and altered lung ventilation, as observed with micro-CT and plethysmography, respectively (Fig 1). During resolution (d12), a population of proliferating BECs appeared in the lungs and this was accompanied by an increased number of venous BECs, while the number of aerocytes did not increase yet. GSEA analysis on aerocytes revealed increased apoptosis, hypoxia and pro-inflammatory pathways at d8 (Fig 5G and S10 Table). At d12, a decrease in pro-inflammatory pathways and an increase in cell cycle-related pathways was observed in the aerocytes, suggesting that these cells are proliferating in an attempt to restore their cell number (Fig 5G). Increased hypoxia and pro-inflammatory pathways were also observed in arterial BECs at d8 and decreased again at d12 (Fig 5H and S11 Table). *Kras* signaling pathway and epithelial mesenchymal transition were detected as increased on d8 in GSEA analysis, which may hint towards an altered morphogenesis and an upregulation of coagulation. In the arterial BECs, increased proliferation and decreased apoptosis were also observed at d12 (Fig 5H). In general, proliferation, based on *Mki67* expression, was increased in almost all nonimmune populations during resolution, confirming that the endothelial and epithelial barrier are being restored (S7B Fig). Despite the limited proportion of other structural cells that could be isolated with the used digestion protocol, we were able to identify different clusters of epithelial cells, fibroblast, mesothelial cells and pericytes. Epithelial cells split into three clusters, type 1 alveolar (AT1), type 2 alveolar (AT2) and ciliated and club epithelial cells (Fig 5A–5C). AT2 cells are mainly important for surfactant production as observed by expression of *Sftpb*, *Sftpd*, *Sftpa* and *Sftpc*, while AT1 express *Cldn18*, *Hopx*, *Akap5* and *Ager*, and are mainly involved in gas exchange (Fig 5C and S9 Table) [20,30]. Some changes were noted in the number of isolated cells from specific epithelial and fibroblast populations, with an apparent increase in AT2 epithelial cells, ciliated epithelial cells, Col13a1^+^ and Col14a1^+^ fibroblasts upon infection at d8, with the number of Col14a1^+^ fibroblasts appearing to remain high at d12 (Fig 5E and 5F). However, it should be noted that the used cell isolation procedure was optimal for leukocytes and endothelial cells, while epithelial cells and fibroblasts may not have been quantitatively isolated. Pericytes are described to surround the capillary endothelium, providing stability, maturation and maintenance of the endothelium [31]. Their number seems not to change upon infection or during resolution (Fig 5E and 5F).

**Fig 6:**
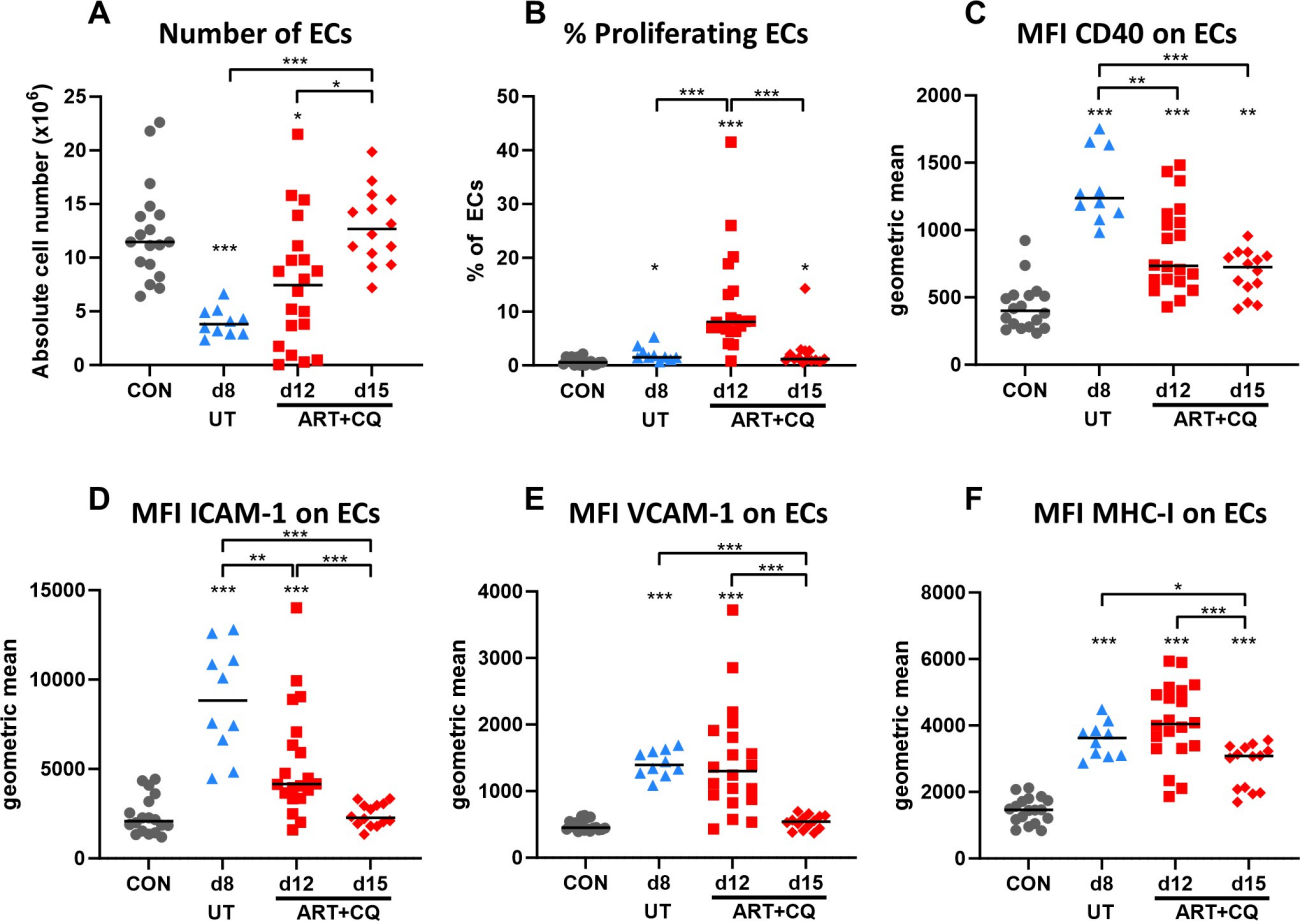
Number of pulmonary endothelial cells decreased upon infection and started to proliferate during resolution to restore their number by 15 dpi accompanied by decreased endothelial activation. *Pb*NK65-infected C57BL/6 mice were treated daily from 8 until 12 dpi with 10 mg/kg artesunate + 30 mg/kg chloroquine (ART+CQ). 2 days and 1 day before sacrifice, mice were injected i.p. with 1.5 mg of bromodeoxyuridine (BrdU). Mice were dissected at 8 dpi for the untreated (UT), *Pb*NK65-infected mice and at 12 or 15 dpi for the ART+CQ-treated, *Pb*NK65-infected C57BL/6 mice. Uninfected mice were used as controls (CON). Cells were isolated from the lungs and flow cytometry was performed. (A) The absolute number of endothelial cells (ECs; CD45^-^ CD31^+^) in the lungs was calculated. (B) Based on BrdU incorporation, the percentage of proliferating ECs was determined. (C-F) The mean fluorescent intensity (MFI) of activation markers, such as CD40 (C), ICAM-1 (D), VCAM-1 (E) and MHC-I (F), on the total ECs was determined. (A-F) Data from two to five experiments. Each symbol represents an individual mouse. Horizontal black lines indicate the median. n = 16–18 for CON, n = 10 for UT d8, n = 20 for ART+CQ d12, n = 14 for ART+CQ d15.

The considerable decrease in BECs upon infection and their restoration during resolution due to proliferation, is a very interesting and novel finding. Therefore, flow cytometry on pulmonary ECs was performed in order to confirm this. In agreement with the scRNAseq data, we observed a drastic decrease in CD31^+^ ECs at 8 dpi (Fig 6A). While in some mice, the number of ECs seemed to be increased again after antimalarial treatment at 12 dpi, in other mice, numbers were still decreased. By 15 dpi, the number of ECs were restored to basal levels. The decrease in CD31^+^ ECs was not due to the downregulation of CD31, since no increase in number of CD45^-^ CD31^-^ cells was observed (S8 Fig). Using BrdU incorporation, a significant increase in BrdU^+^ ECs was observed at 12 dpi, suggesting that ECs are proliferating during the resolution phase in order to restore their absolute number by 15 dpi (Fig 6B). In addition, at 8 dpi, ECs were found to be activated, as observed with an increased CD40 expression (Fig 6C), and upregulated the expression of adhesion molecules, such as intercellular adhesion molecule-1 (ICAM-1; Fig 6D) and vascular cell adhesion molecule-1 (VCAM-1; Fig 6E), and of major histocompatibility complex I (MHCI-I; Fig 6F). At 12 dpi, CD40 and ICAM-1 expression on ECs was decreased already, while VCAM-1 and MHC-I only decreased at 15 dpi (Fig 6C–6F). But, even at 15 dpi, CD40 and MHC-I on ECs was still increased in comparison to baseline level, suggesting persistent EC activation (Fig 6C–6F).

Overall, these data show a drastic decrease of microvascular EC numbers during infection. This is consistent with the notion of CD8^+^ cytotoxic T cells targeting the endothelium as major driver of the MA-ARDS pathology. Furthermore, the proliferation of the endothelial cells on day 12 suggest that this is a major event in the restauration of the pulmonary integrity.

### Cell-to-cell communication regulating the dynamics in the lung during MA-ARDS and its resolution

Information obtained during the subcluster analyses were integrated into the original analysis containing all cells (S9A Fig) and used to identify interesting ligand-receptor interactions and their potential targets between cells in order to establish a cell-cell communication network [32]. Some subclusters were combined to obtain larger clusters per sample for the MultiNicheNet analysis with the goal of obtaining more robust results and to avoid the exclusion of certain clusters due to low numbers (S9B Fig).

**Fig 7:**
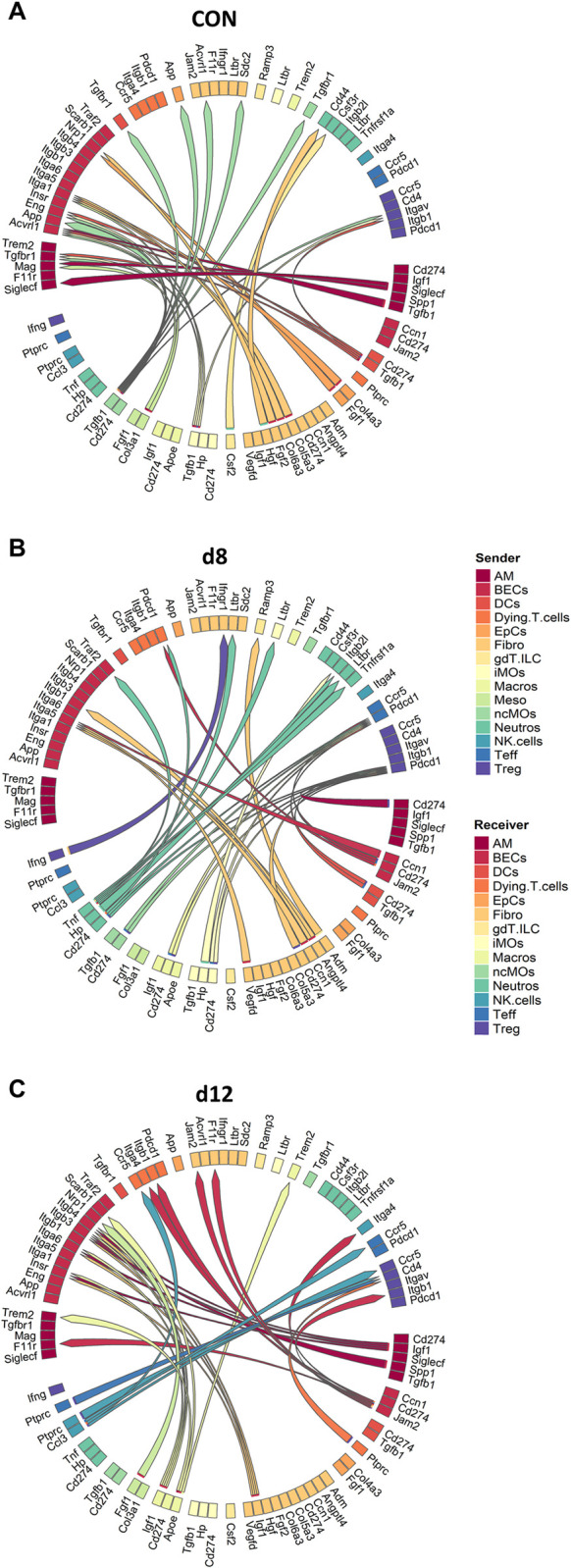
Overview of the pulmonary cell-cell communication in uninfected mice, mice with MA-ARDS and mice recovering from MA-ARDS. Interactome analysis was performed on the original lung scRNAseq analysis of control (CON), *Pb*NK65-infected (d8) and ART+CQ-treated (d12) mice using the MultiNicheNet package. (A-C) Circos plots showing the top 30 ligand-receptor interactions per condition compared to the other two conditions for CON (A), d8 (B) and d12 (C), determined using MultiNicheNet. Subclusters were combined into bigger clusters to make the results more robust (S5 Fig). All clusters were selected as senders and all as receivers. (A-C) n = 4 for CON, d8 and d12 (4 mice per condition were pooled using Hashtag oligos). AM, alveolar macrophages; BECs, blood endothelial cells; DCs, dendritic cells; EpCs, epithelial cells; Fibro, fibroblasts; gdT.ILC, γδ T cells/innate lymphoid cells; iMOs, inflammatory monocytes; Macros, macrophages; Meso, mesothelial cells; ncMOs, non-classical monocytes; Neutros, neutrophils; NK.cells, natural killer cells; Teff, effector T cells; Treg, regulatory T cells.

In uninfected controls, the top 30 cell-cell interactions appeared to involve signaling via TGF-β (*Tgfb1*), an immunoregulatory and pro-fibrotic cytokine, and fibroblast growth factor 1 (*Fgf1*) and 2 (*Fgf2*), which are pro-angiogenic factors (Fig 7A) [33–35]. In addition, epithelial cells, fibroblasts and mesothelial cells expressed genes inducing collagen production (*Col4a3*, *Col5a3*, *Col6a3*, *Col3a1*). MultiNicheNet analysis revealed a potential interaction of hepatocyte growth factor (*Hgf)* and colony stimulating factor 2 (*Csf2)* from fibroblasts and γδ T cells/ILCs with neutrophils, which could be involved in the promotion of neutrophil survival [36,37]. In the literature, the lung has been described as a neutrophil reservoir during steady state [38]. AM appeared to communicate with other AM via SiglecF to fine-tune their activation and prevent unnecessary immune responses [39].

Upon infection (d8), Programmed cell death ligand 1 (PD-L1; *Cd274*) on AM, BECs, DCs, Fibro, iMOs, Macros, ncMOS and Neutros appear to interact with Programmed cell death protein 1 (PD-1; *Pdcd1*) on Teff, Treg cells and dying T cells, in an attempt to inhibit T cell proliferation, cytokine production and cytotoxicity (Fig 7B) [40,41]. In addition, *Tnf* and *Ifng* signaling was found confirming the pro-inflammatory state of the lungs at d8. Adrenomedullin (*Adm*) was found to induce ILC2 activation [42]. *Vegfd*, *Angptl4* and *Ccn1* interact with integrins on the EC surface, thereby inducing angiogenesis in an attempt to correct for the decreased number of ECs [43–45].

At d12, insulin-like growth factor 1 (*Igf1*) was found in AM, Fibro and Macros and interacted with different integrins (*Itgb3*, *Itgb4* and *Itga6*) and the insulin receptor (*Insr*) (Fig 7C). In the literature, this interaction has been described to induce vasodilation, possibly via the production of nitric oxide, and to promote angiogenesis [46–48]. In addition, as in uninfected controls, interaction was found between *Fgf1* and *Nrp1*, which may also induce angiogenesis. *Jam2* on BECs may play an important role in leukocyte transmigration, via binding to integrins, or in endothelial junctions, via binding to *F11r* or itself.[49,50] Lymphoid cells, such as NK cells, Teff cells and dying T cells were found to play a role in the activation of Treg cells via interaction of CD45 (*Ptprc*) with CD4 [51–53]. Moreover, NK cells express *Ccl3*, which may promote CD8^+^ T cell activation via the CCR5 receptor [54]. *Apoe* promotes the M2 macrophage phenotype and may be involved in promoting survival of macrophages and regulate phagocytosis [55–58]. *Fgf1* was also found at d12 as in uninfected controls to bind to Neuropilin 1 (*Nrp1*) on BECs which may induce angiogenesis [34,35].

These results suggest an increased inflammatory state in the lungs at d8 and upregulation of anti-inflammatory pathways and promotion of proliferation of BECs at d12.

**Fig 8:**
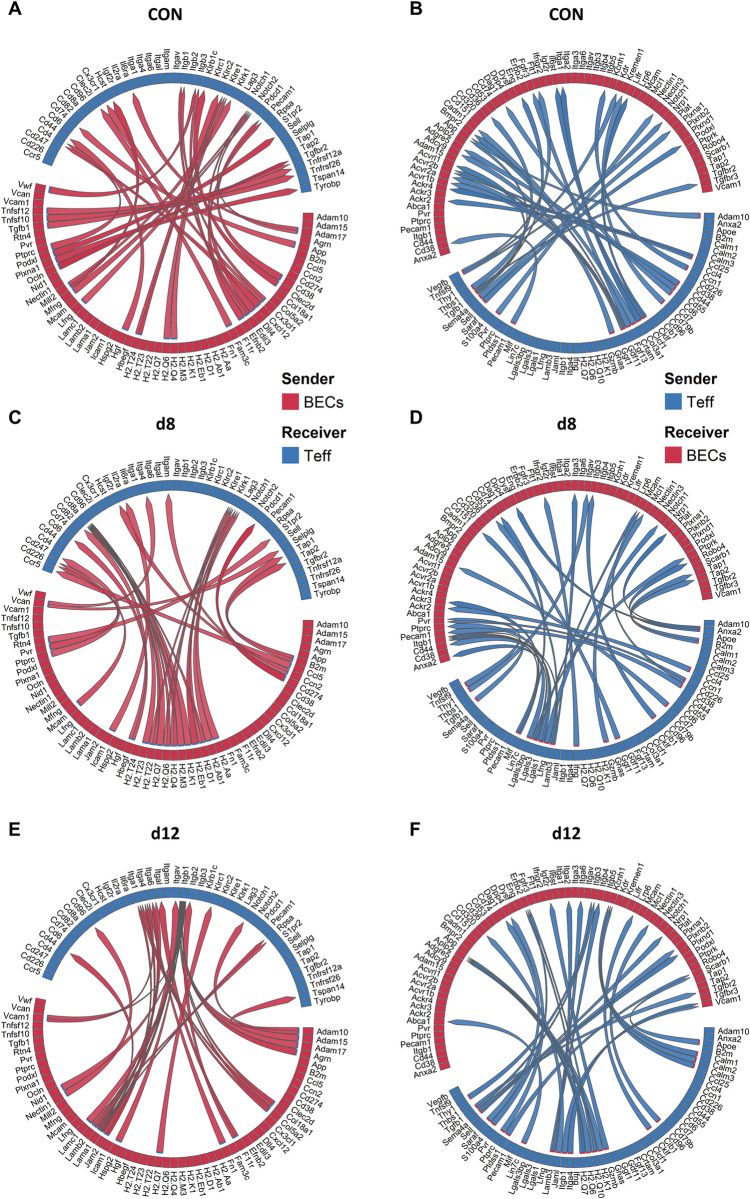
Pulmonary cell-cell communication between blood endothelial cells and effector T cells. Interactome analysis was performed on the original lung scRNAseq analysis of control (CON), *Pb*NK65-infected (d8) and ART+CQ-treated (d12) mice using the MultiNicheNet package with blood endothelial cells (BECs) as sender and effector T cells (Teff) as receiver (A,C,E) or with Teff cells as sender and BECs as receivers (B, D, F). Circos plots showing the top 30 ligand-receptor interactions per condition compared to the other two conditions for CON (A,B), d8 (C,D) and d12 (E,F) determined using MultiNicheNet. Subclusters were combined into bigger clusters to make the results more robust (S5 Fig). n = 4 for CON, d8 and d12 (4 mice per condition were pooled using Hashtag oligos. BECs, blood endothelial cells; Teff, effector T cells.

Because Claser *et al*. described the killing of ECs by CD8^+^ T cells in MA-ARDS, the top 30 cell-cell interactions between the BECs and Teff cells was studied (4). In uninfected controls, signaling via TGF-β (*Tgfb1*) was again found both when investigating interactions between BECs as sender and Teff as receiver (Fig 8A) and with Teff as sender and BECs as receiver (Fig 8B). However, in case of BECs as receiver, *Tgfb1* interacted with *Tgfbr3*, which does not have kinase activity. Other interactions between BECs and Teff included Notch signaling, Killer-like lectin receptors, Cx3cr1 and multiple integrins (Fig 8A and 8B).

Upon infection, signaling of BECs via MHC-related genes to *Cd8a* and Killer-like lectin receptors was found by MultiNicheNet analysis, indicative of antigen presentation by BECs in MHC-I to the Teff cells (Fig 8C). Moreover, *IFNg* and *Gzmb* were found on the Teff cells as ligands to the BECs, suggesting that Teff cells are indeed activated and may therefore kill the BECs (Fig 8D). In an attempt to balance the pro-inflammatory response, PD-L1 (*Cd274*)–PD-1 (*Pdcd1*) interaction between the Teff and BECs was also found on d8 (Fig 8C and 8D). At day 12, interaction of BECs with Teff via MHC-related genes decreased (Fig 8E). During resolution multiple Calmodulin genes (*Calm1*, *Calm2*, Calm3) were expressed by Teff cells which interact with *Kcnh1*, a gene coding for a potassium channel that may be important in proliferation, on the BECs (Fig 8F). Our scRNAseq data are thus in-line with the notion of an interaction between BECs and Teff cells upon infection, with BECs presenting antigens in a MHC context, resulting in activation of the Teff cells and subsequent expression of *Ifng* and *Gzmb*.

**Fig 9:**
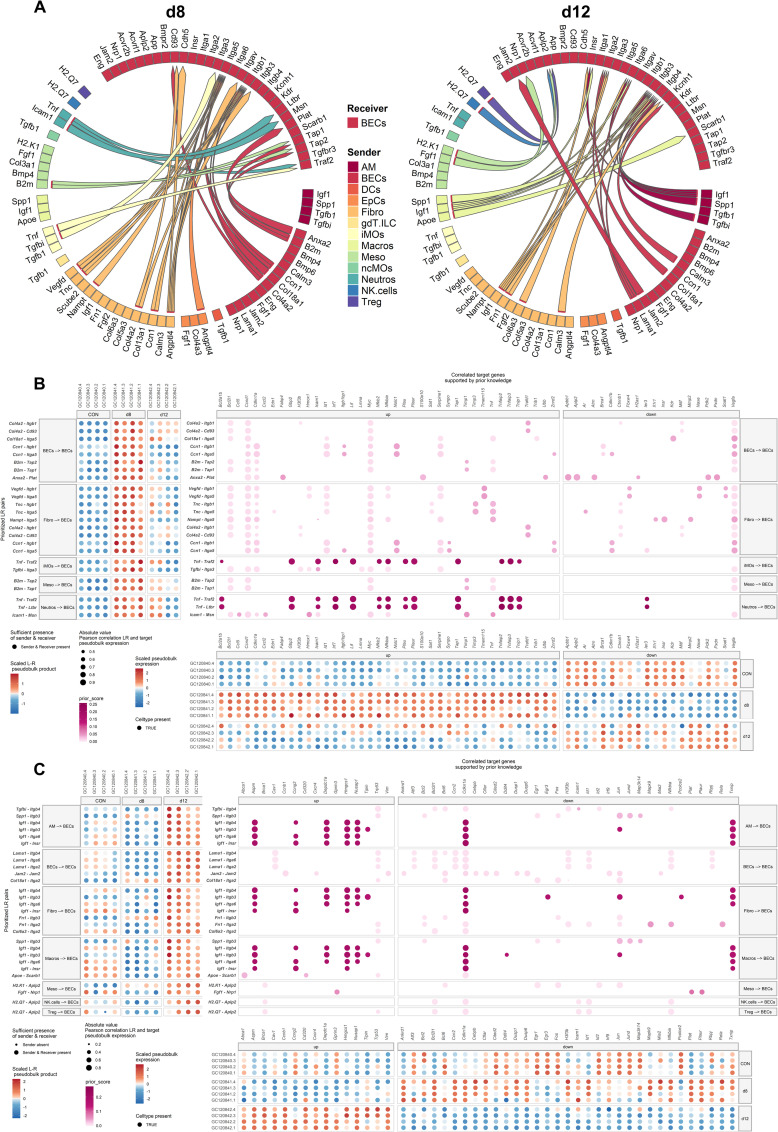
Pulmonary cell-cell communication with blood endothelial cells as receivers. Interactome analysis was performed on the original lung scRNAseq analysis of control (CON), *Pb*NK65-infected (d8) and ART+CQ-treated (d12) mice using the MultiNicheNet package with only blood endothelial cells as receivers. (A) Circos plots showing the top 30 ligand-receptor interactions per condition compared to the other two conditions for d8 (left panel) and d12 (right panel), determined using MultiNicheNet. Subclusters were combined into bigger clusters to make the results more robust (S5 Fig). All clusters were selected as senders and blood endothelial cells as receivers. (B-C) Correlation plot showing both ligand-receptor pair and predicted target gene expression were visualized with the predicted target ligand-target links at d8 (B) and d12 (C). (A-C) n = 4 for CON, d8 and d12 (4 mice per condition were pooled using Hashtag oligos). AM, alveolar macrophages; BECs, blood endothelial cells; DCs, dendritic cells; EpCs, epithelial cells; Fibro, fibroblasts; gdT.ILC, γδ T cells/innate lymphoid cells; iMOs, inflammatory monocytes; Macros, macrophages; Meso, mesothelial cells; ncMOs, non-classical monocytes; Neutros, neutrophils; NK.cells, natural killer cells; Teff, effector T cells; Treg, regulatory T cells.

The increased proliferation of endothelial cells that was observed during the resolution phase (Fig 6B), was further investigated since promotion of this proliferation process may be beneficial during the resolution process. Therefore, cell-cell communication that may be involved in these processes was investigated by evaluating the top 30 cell-cell interaction with BECs as receiver. In addition, the ligand activities for a sender-receiver combination, the predicted ligand-target links and the expression of the predicted target genes were determined across samples with BECs as receiver. At d8, cell-cell communication with BECs as receiver mainly involves pro-inflammatory molecules, such as *Tnf*, *Icam1* and *B2m*, pro-angiogenic factors, such as *Vegfd*, *Ccn1* and *Angptl4*, and pro-fibrotic factors, such as *Col18a1*, *Col4a2*, *Col13a1* and *Col4a2* (Fig 9A, left panel). As shown in Fig 9B, the ligand-receptor pairs with the highest prioritization score are Tnf, expressed by iMOs and neutrophils, interacting with BECs. This may be involved in inducing the expression of downstream targets involved in angiogenesis, both positive (*Irf7*, *Plau*) and negative (*Gbp2*, *Lif*) regulation, positive regulation of coagulation (*Plau*, *Plaur*) and in inflammation, with also both pro- (*Icam1*, *Irf7*, *Nfkb2*, *Nfkbia*, *Tap1*) and anti-inflammatory signals (*Bcl2a1b*, *Tnfaip2*, *Tnfaip3*, *Tnip1*) [29,59–69]. At d12, AMs, Macros and fibroblasts express insulin-like growth factor (*Igf1)* and mesothelial cells express fibroblast growth factor 1 (*Fgf1)* which both promote angiogenesis (Fig 9A, right panel). This was confirmed by the downstream targets of *Igf1* interaction with integrins and the insulin receptor (Insr) (Fig 9C). Almost all target genes were related to cell cycle progression or proliferation (*Aspm*, *Depdc1a*, *Nusap1*, *Tipin*, *Cdkn1a*, *Ddit4*, *Egr3*) [70–76]. Interaction of *Apoe* with *Scarb1* on BECs induced *Abca1* expression, which is an anti-inflammatory gene (Fig 9A, right panel and 9C) [77]. Osteopontin (*Spp1*) was found to be expressed during resolution by macrophages and interacts with similar integrins as insulin-like growth factor 1 (Fig 9A, right panel and 9C). Osteopontin (*Spp1*) and laminin subunit alpha 1 (*Lama1*) had *Trp53* as potential target gene in the BECs, which is described to be involved in EC senescence (Fig 9C) [78].

Investigating downstream signaling targets of the obtained ligand-receptor interaction with BECs as receiver, confirmed the highly inflammatory state upon infection and the promotion of proliferation of BECs and anti-inflammatory genes during resolution. Overall, this interactome analysis based on the single cell transcriptomes generates a broad and detailed overview of the major processes and pathways occurring during pathology and resolution in experimental MA-ARDS. Furthermore, it provides a highly useful roadmap for further investigation of possible targets to enhance disease resolution.

## Discussion

In this paper, we combined functional assessment of lung alterations and lung ventilation together with analysis of single cell transcriptomics to define mechanisms involved in the development and resolution of MA-ARDS. Micro-CT revealed an increase in total lung volume and non-aerated lung volume, which is caused by edema development and inflammation, resulting in a decreased breathing frequency and tidal volume and thus a drastic decrease in minute volume, which reflects the potential for gas exchange. In addition, an increase in end expiratory pause and enhanced pause was observed, also contributing to the decreased frequency. The increased total lung volume can be attributed to a compensatory mechanism of mice in an attempt to prevent loss of airspace due to increased disease [79,80]. This compensatory mechanism may explain the increased aerated lung volume at 6 dpi, since the total lung volume already displayed a trend towards an increased volume while no edema is present yet. From 8 dpi onwards, increased non-aerated lung volume was observed, corresponding with the presence of edema. This edema may be responsible for the decrease in tidal volume upon infection. The compensatory mechanism appears to avoid a decrease in aerated lung volume despite the presence of edema. This enlarged state of the lungs limits the capacity for further expansion of the lungs needed for inspiration, hence leading to a lower tidal volume and thus lower respiratory capacity. As shown in the literature, an increased elastance and decreased compliance has been described upon infection [5,81]. This may also cause a decrease in passive expiration thereby contributing to an increased end expiratory pause and therefore decreased breathing frequency and thus minute volume. During resolution upon antimalarial treatment, edema was found to be cleared resulting in restoration of lung ventilation parameters.

ScRNAseq analysis confirmed a highly inflammatory state of the lungs upon *Pb*NK65 infection, with activation of different leukocyte populations, such as T cells, NK cells and AM. In general, changes in the number of lymphoid and myeloid cell populations obtained with scRNAseq, confirmed our previously obtained data on pulmonary lymphoid and myeloid cell numbers using flow cytometry [13]. The procedure used to isolate cells from the lungs for flow cytometry or scRNAseq appeared to be optimal for the isolation of leukocytes and endothelial cells, while the retrieval of other non-immune cell populations, such as epithelial cells and fibroblasts, was limited, thereby making strong conclusions on these populations more difficult. Moreover, a decrease in endothelial cells, especially aerocytes and venous ECs, was observed in the scRNAseq analysis and this was confirmed using flow cytometry with a specific panel to detect ECs. The activation of cytotoxic CD8^+^ T cells and decreased number of ECs upon infection, corroborate the findings of Claser *et al*. that cytotoxic CD8^+^ T cells are responsible for pulmonary edema development by killing parasite antigen-presenting ECs [4]. Sercundes *et al*. also demonstrated increased apoptosis of ECs in a mouse model of MA-ARDS [82]. Also in mouse models of ECM, apoptosis of ECs was observed [83–86]. In a patient study, increased EC apoptosis was found to be associated with lethal CM [87]. Most interestingly, Dorovini-Zis *et al*. found hypertrophic ECs with prominent vesicular nuclei, endothelial necrosis and even vessels denuded of endothelium in CM [88]. Moreover, Punsawad *et al*. demonstrated that the Fas/FasL system induced activation of caspases which mediate apoptosis of ECs and may therefore contribute to pulmonary edema in patients [89]. Also in *in vitro* cultures of human ECs with infected red blood cells, an apoptotic effect on ECs was observed [90,91]. In contrast, in some studies, permeabilization of the ECs rather than apoptosis of the ECs was described to be involved in ECM pathogenesis [92,93]. In these studies, disassembly of tight and adherens junctions which affect fluid transport was found to be responsible for pathology. In addition, Shaw *et al*. found only limited apoptosis in ECM development [94]. Taken together, there is conflicting data on whether the disruption of the endothelial barrier is caused by death of the ECs or only a permeabilization of the ECs without death. Our results clearly corroborate the idea that death of the ECs is responsible for the development of edema. Aerocytes are the endothelial cells that align the alveolar epithelium and are important in gas exchange [19,20]. Their depletion upon infection and the development of edema may result in reduced gas exchange capacity. Indeed, we and others have described a decreased gas exchange and increased CO_2_ levels in the blood, consistent with MA-ARDS pathology [95,96].

GSEA analysis on multiple leukocyte populations revealed an increase in pro-resolving and anti-inflammatory pathways being active during the resolution phase of MA-ARDS. This was accompanied by an increased proliferation of ECs, as shown with both scRNAseq and flow cytometry, with the number of pulmonary ECs back at basal level by 15 dpi. Treatments that may prevent apoptosis or killing of the ECs upon infection or promote the proliferation of ECs may be interesting as adjunctive therapies in combination with antimalarial drugs to ameliorate the resolution of MA-ARDS. Moreover, persistent EC activation was observed with elevated levels of CD40 and MHC-I on the ECs until 15 dpi. This is in agreement with a clinical study of Moxon *et al*., who demonstrated persistent endothelial activation and inflammation in children with cerebral malaria until one week after admission to the hospital.

CD8^+^ T cells were found to be pathogenic in experimental MA-ARDS and ECM [4,6,15,97]. Also in pediatric patients, cerebral malaria was found to be associated with cerebrovascular engagement of CD3^+^ CD8^+^ T cells [98]. Therefore, these results suggest that both in ECM and experimental MA-ARDS, endothelial cells direct CD8^+^ T cell recruitment and activation in the brain or lung, respectively, causing pathology. Claser *et al*. and Howland *et al*. demonstrate cross-presentation of parasite antigens via MHC-I on endothelial cells causing activation of the CD8^+^ T cells and subsequently damage to the vascular wall in both experimental MA-ARDS and cerebral malaria (ECM) respectively [4,83]. In our scRNAseq analysis in MA-ARDS, this was confirmed by cell-cell interaction predictions between the BECs and Teff cells. This suggests that BECs present antigens to Teff cells via MHC, resulting in the activation of the Teff cells and the subsequent expression of *Ifng* and *Gzmb* (Fig 8). Moreover, we observed that these interactions were mainly specific for d8, since the interactions were no longer present at d12. This is in agreement as well with Claser *et al*., who demonstrated a decrease in cross-presentation by pulmonary endothelial cells upon antimalarial treatment [4]. Together with the finding that most cytokines are downregulated on d12 compared to d8 [13], these data provide insight in why disease symptoms are already broadly resolving on d12, despite the even higher number of pathogenic CD8^+^ T cells on d12 compared to d8.

Cell-cell communication analysis with the BECs as receiver (Fig 9) could suggest that anti-TNF therapy may prevent excessive inflammation and affect angiogenesis. However, a clinical trial using anti-TNF antibodies against cerebral malaria found no effect on mortality and even increased neurologic sequelae in children that received the antibody treatment [99,100]. Pentoxifylline treatment, which inhibits TNF-α production, was also tested in clinical studies, but no major improvements of clinical parameters or survival were observed, except for some studies showing a decreased coma duration [99]. Although these studies were performed with cerebral malaria and not MA-ARDS, they suggest that blocking TNF pathways may not be the best option to promote recovery from MA-ARDS.

Promoting resolution and recovery, for example by promoting proliferation of ECs, may be more beneficial. In general, pro-resolving therapies have been described to have less side-effects than anti-inflammatory therapies [101]. Based on the MultiNicheNet analysis at d12, insulin-like growth factor 1 (IGF-1) may be an interesting target to promote the resolution of MA-ARDS, since IGF-1 induced cell cycle- and proliferation-related target genes in the BECs. Moreover, IGF-1 production by AM has also been described to induce proliferation and differentiation of type 2 alveolar (AT2) epithelial cells and subsequently increase repair of airway epithelium and gas exchange [8,102,103]. Osteopontin (*Spp1*) was also found to be expressed during resolution by macrophages and interacts with similar integrins as IGF-1. Osteopontin is described to reduce inflammation and decrease tissue injury and may therefore be an interesting target as well.[104] Apolipoprotein E (*Apoe*) may have anti-inflammatory effects in BECs by inducing ATP binding Cassette Subfamily A Member 1 (*Abca1)* [77]. Moreover, *Apoe* was found to suppress T cell activation and promote the M2 macrophage phenotype [24–26,55–58]. Taken together all effects of *Apoe* on BECs, T cells and macrophages, induction of *Apoe* may promote resolution of inflammation. Further investigations to functionally validate these different resolution pathways are underway.

In conclusion, micro-CT revealed an increase in total lung volume and non-aerated lung volume upon infection with *Pb*NK65, which is caused by edema development. This resulted in a drastically altered lung ventilation pattern, which was restored during the resolution of MA-ARDS after parasite killing. Besides the highly inflammatory state in the lungs, scRNAseq revealed a considerable decrease in endothelial cell numbers. During the resolution phase, ECs started to proliferate in order to restore the endothelial barrier. An increase in anti-inflammatory pathways was also observed during resolution. These findings serve as an interesting starting point to investigate adjunctive treatments that promote pro-resolving pathways that limit inflammation and treatments that induce proliferation of ECs, since these may improve the recovery from the otherwise lethal MA-ARDS complication after parasite killing with antimalarial drugs.

## Supporting information

S1 Fig Flow cytometry gating strategy for endothelial cells. Lung cells were isolated and stained for flow cytometry. After exclusion of red blood cells, debris and doublets of cells, all live cells (LD^-^) were gated. The endothelial cells were identified as CD45^-^ CD31^+^. BrdU and activation markers were analyzed on the endothelial cells (both frequency of positive cells and mean fluorescent intensity). Representative gatings of an uninfected control and of an ART+CQ-treated, *Pb*NK65-infected C57BL/6 mouse at 12 dpi is shown. LD, live dead.
(TIF)

S2 Fig Follow-up of ART+CQ-treated, *Pb*NK65-infected C57BL/6 mice. *Pb*NK65-infected C57BL/6 mice were treated daily from 8 until 12 dpi with 10 mg/kg artesunate + 30 mg/kg chloroquine (ART+CQ). (A) Parasitemia was determined daily starting at 6 dpi using Giemsa-stained blood smears. (B) Clinical score was monitored daily starting at 6 dpi. (C) Body weight loss was calculated compared to 0 dpi starting at 6 dpi. (A-C) Data from three experiments. Data are represented as means ± SEM. n = 10 for CON, n = 15–18 for ART+CQ.
(TIF)

S3 Fig Level of alveolar edema in mice selected for scRNAseq analysis. *Pb*NK65-infected C57BL/6 mice were treated daily from 8 until 12 dpi with 10 mg/kg artesunate + 30 mg/kg chloroquine (ART+CQ). Level of alveolar edema was determined by measuring the protein concentration in the BALF. Four mice (full symbols) per condition were selected from the experiment to perform scRNAseq.
(TIF)

S4 Fig Expression levels of *GzmB*, *Ccl5* and *Ccl4* in pulmonary lymphoid cell populations. Lymphoid cell clusters (Fig 3) from control (CON), *Pb*NK65-infected (d8) and ART+CQ-treated (d12) mice were checked for the expression of different genes. Expression levels of *Granzyme B* (*GzmB*; A), *CC chemokine ligand 5* (*Ccl5*; B) and *CC chemokine ligand 4* (*Ccl4*; C) in the different lymphoid cell populations in all three conditions are shown.
(TIF)

S5 Fig Doublet1 and Doublet2 cluster separately from other pulmonary lymphoid cells. Doublet1 and Doublet2 cluster were identified in the myeloid analysis (Fig 4) with T cell or B cell markers as marker genes respectively. Therefore, these clusters were added to the lymphoid cells (Fig 3) and clustered again. (A) UMAP plot showing where the Doublet1 and Doublet2 clusters are located. (B) UMAP plot according to the major cell types with Doublet1 and Doublet2 clustering together as a myeloid cell cluster. (C) Heatmap displaying the top 5 markers of each cell type and top 15 of the myeloid cluster (from B) and its expression across all cell types.
(TIF)

S6 Fig Expression levels of *Alox15*, *Hmox1* and *Fth1* in pulmonary myeloid cell populations. Myeloid cell clusters (Fig 4) from control (CON), *Pb*NK65-infected (d8) and ART+CQ-treated (d12) mice were checked for the expression of different genes. Expression levels of *15-lipoxygenase* (*Alox15*; A), *Heme oxygenase 1* (*Hmox1;* B) and *Ferritin heavy chain 1* (*Fth1;* C) in the different myeloid cell populations in all three conditions are shown.
(TIF)

S7 Fig Expression levels of *Vegfa* and *Mki67* in pulmonary nonimmune cell populations. Non-immune cell clusters (Fig 5) from control (CON), *Pb*NK65-infected (d8) and ART+CQ-treated (d12) mice were checked for the expression of different genes. Expression levels of *vascular endothelial growth factor* (*Vegfa*; A) and *Marker of proliferation Ki-67* (*Mki67;* B) in the different nonimmune cell populations in all three conditions are shown.
(TIF)

S8 Fig No increase in CD45^-^ CD31^-^ cells was observed upon *Pb*NK65 infection. *Pb*NK65-infected C57BL/6 mice were treated daily from 8 until 12 dpi with 10 mg/kg artesunate + 30 mg/kg chloroquine (ART+CQ). Mice were dissected at 8 dpi for the untreated (UT), *Pb*NK65-infected mice and at 12 or 15 dpi for the ART+CQ-treated, *Pb*NK65-infected C57BL/6 mice. Uninfected mice were used as controls (CON). Cells were isolated from the lungs and flow cytometry was performed. (A) Representative FACS plots showing CD45 and CD31 expression on all live single cells, with ECs (CD45^-^ CD31^+^) and leukocytes (CD45^+^ CD31^-^) gated in black and CD45^-^ CD31^-^ population in orange. (B) The absolute number of CD45^-^ CD31^+^ in the lungs was calculated. Data from two to five experiments. Each symbol represents an individual mouse. Horizontal black lines indicate the median. n = 16–18 for CON, n = 10 for UT d8, n = 20 for ART+CQ d12, n = 14 for ART+CQ d15.
(TIF)

S9 Fig Information of subclustering is projected onto original UMAP plot. (A) Subcluster identification obtained during the subclustering of lymphoid, myeloid and nonimmune cells, was projected onto the UMAP plot of the original analysis (Fig 2A). (B) Some subclusters were combined into bigger populations for the MultiNicheNet analysis to make the results more robust. UMAP plot of the original analysis showing the clusters used in the MultiNicheNet analysis.
(TIF)

S1 Table Antibodies used for flow cytometry. Table listing an overview of the antibodies used for flow cytometry including the antigen, fluorophore and the company. 200 000 live single cells were read for each sample.
(DOCX)

S2 Table Marker genes for the main clusters. List of marker genes that were identified using the FindAllMarkers function for all main clusters as shown in Fig 2. Table contains the gene name (rowname and gene), the p-value (p_val), the average log2 fold change compared to all other clusters (avg_log2FC), percent of cells expressing the gene in cluster of interest (pct.1), percent of cells expressing the genes in all other clusters (pct. 2), adjusted p-value (p_val_adj) and the cluster to which the gene belongs (cluster).
(XLSX)

S3 Table Marker genes for the lymphoid clusters. List of marker genes that were identified using the FindAllMarkers function for all lymphoid clusters as shown in Fig 3. Table contains the gene name (rowname and gene), the p-value (p_val), the average log2 fold change compared to all other clusters (avg_log2FC), percent of cells expressing the gene in cluster of interest (pct.1), percent of cells expressing the genes in all other clusters (pct. 2), adjusted p-value (p_val_adj) and the cluster to which the gene belongs (cluster)
(XLSX)

S4 Table Output of the GSEA analysis for NK cells. Overview of the hallmark pathways, their p-value (pval), adjusted p-value (padj), enrichment score (ES), normalised enrichment score (NES), number of times a random gene set had a more extreme enrichment score value (nMoreExtreme), size of the pathway after removing genes not present in list of differentially expressed genes (size) and a vector with indexes of leading edge genes that drive the enrichment (leadingEdge) when comparing the different conditions (CONvsd8, CONvsd12 and d8vsd12) for the NK cells as shown in Fig 3G.
(XLSX)

S5 Table Output of the GSEA analysis for T cells. Overview of the hallmark pathways, their p-value (pval), adjusted p-value (padj), enrichment score (ES), normalised enrichment score (NES), number of times a random gene set had a more extreme enrichment score value (nMoreExtreme), size of the pathway after removing genes not present in list of differentially expressed genes (size) and a vector with indexes of leading edge genes that drive the enrichment (leadingEdge) when comparing the different conditions (CONvsd8, CONvsd12 and d8vsd12) for all T cells combined as shown in Fig 3H.
(XLSX)

S6 Table Marker genes for the myeloid clusters. List of marker genes that were identified using the FindAllMarkers function for all myeloid clusters as shown in Fig 4. Table contains the gene name (rowname and gene), the p-value (p_val), the average log2 fold change compared to all other clusters (avg_log2FC), percent of cells expressing the gene in cluster of interest (pct.1), percent of cells expressing the genes in all other clusters (pct. 2), adjusted p-value (p_val_adj) and the cluster to which the gene belongs (cluster).
(XLSX)

S7 Table Output of the GSEA analysis for alveolar macrophages. Overview of the hallmark pathways, their p-value (pval), adjusted p-value (padj), enrichment score (ES), normalised enrichment score (NES), number of times a random gene set had a more extreme enrichment score value (nMoreExtreme), size of the pathway after removing genes not present in list of differentially expressed genes (size) and a vector with indexes of leading edge genes that drive the enrichment (leadingEdge) when comparing the different conditions (CONvsd8, CONvsd12 and d8vsd12) for the alveolar macrophages as shown in Fig 4G.
(XLSX)

S8 Table Output of the GSEA analysis for monocytes. Overview of the hallmark pathways, their p-value (pval), adjusted p-value (padj), enrichment score (ES), normalised enrichment score (NES), number of times a random gene set had a more extreme enrichment score value (nMoreExtreme), size of the pathway after removing genes not present in list of differentially expressed genes (size) and a vector with indexes of leading edge genes that drive the enrichment (leadingEdge) when comparing the different conditions (CONvsd8, CONvsd12 and d8vsd12) for all monocytes as shown in Fig 4H.
(XLSX)

S9 Table Marker genes for the non-immune clusters. List of marker genes that were identified using the FindAllMarkers function for all non-immune clusters as shown in Fig 5. Table contains the gene name (rowname and gene), the p-value (p_val), the average log2 fold change compared to all other clusters (avg_log2FC), percent of cells expressing the gene in cluster of interest (pct.1), percent of cells expressing the genes in all other clusters (pct. 2), adjusted p-value (p_val_adj) and the cluster to which the gene belongs (cluster).
(XLSX)

S10 Table Output of the GSEA analysis for aerocytes. Overview of the hallmark pathways, their p-value (pval), adjusted p-value (padj), enrichment score (ES), normalised enrichment score (NES), number of times a random gene set had a more extreme enrichment score value (nMoreExtreme), size of the pathway after removing genes not present in list of differentially expressed genes (size) and a vector with indexes of leading edge genes that drive the enrichment (leadingEdge) when comparing the different conditions (CONvsd8, CONvsd12 and d8vsd12) for the aerocytes as shown in Fig 5G.
(XLSX)

S11 Table Output of the GSEA analysis for arterial blood endothelial cells. Overview of the hallmark pathways, their p-value (pval), adjusted p-value (padj), enrichment score (ES), normalised enrichment score (NES), number of times a random gene set had a more extreme enrichment score value (nMoreExtreme), size of the pathway after removing genes not present in list of differentially expressed genes (size) and a vector with indexes of leading edge genes that drive the enrichment (leadingEdge) when comparing the different conditions (CONvsd8, CONvsd12 and d8vsd12) for all arterial blood endothelial cells as shown in Fig 5H.
(XLSX)
