# Supplementary figures and images for: Single cell RNA sequencing reveals endothelial cell killing and resolution pathways in experimental malaria-associated acute respiratory distress syndrome
Source: PLoS Pathog. 2024 Jan 18;20(1):e1011929. doi: 10.1371/journal.ppat.1011929 (PMC10826972; doi:10.1371/journal.ppat.1011929)

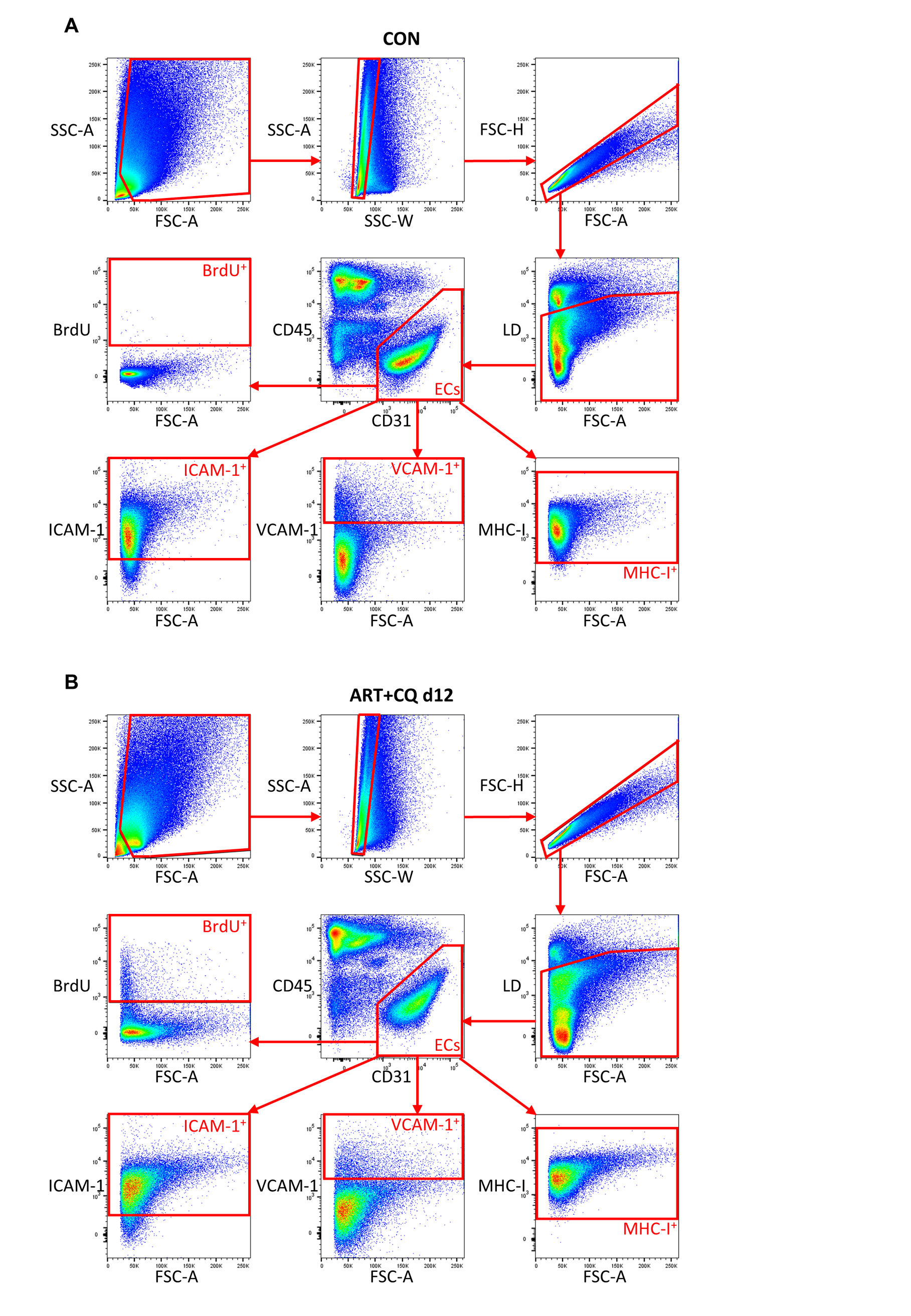

Supplement: S1 Fig — Lung cells were isolated and stained for flow cytometry. After exclusion of red blood cells, debris and doublets of cells, all live cells (LD-) were gated. The endothelial cells were identified as CD45- CD31+. BrdU and activation markers were analyzed on the endothelial cells (both frequency of positive cells and mean fluorescent intensity). Representative gatings of an uninfected control and of an ART+CQ-treated, PbNK65-infected C57BL/6 mouse at 12 dpi is shown. LD, live dead. (TIF) [file ppat.1011929.s001.tif]

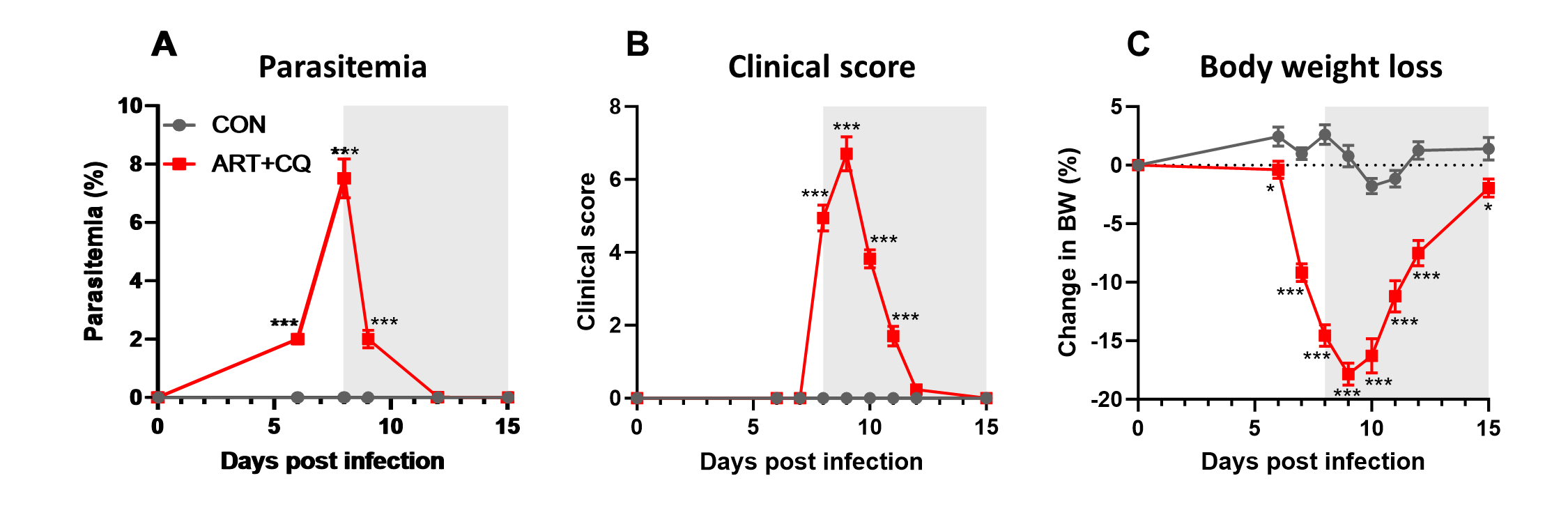

Supplement: S2 Fig — PbNK65-infected C57BL/6 mice were treated daily from 8 until 12 dpi with 10 mg/kg artesunate + 30 mg/kg chloroquine (ART+CQ). (A) Parasitemia was determined daily starting at 6 dpi using Giemsa-stained blood smears. (B) Clinical score was monitored daily starting at 6 dpi. (C) Body weight loss was calculated compared to 0 dpi starting at 6 dpi. (A-C) Data from three experiments. Data are represented as means ± SEM. n = 10 for CON, n = 15–18 for ART+CQ. (TIF) [file ppat.1011929.s002.tif]

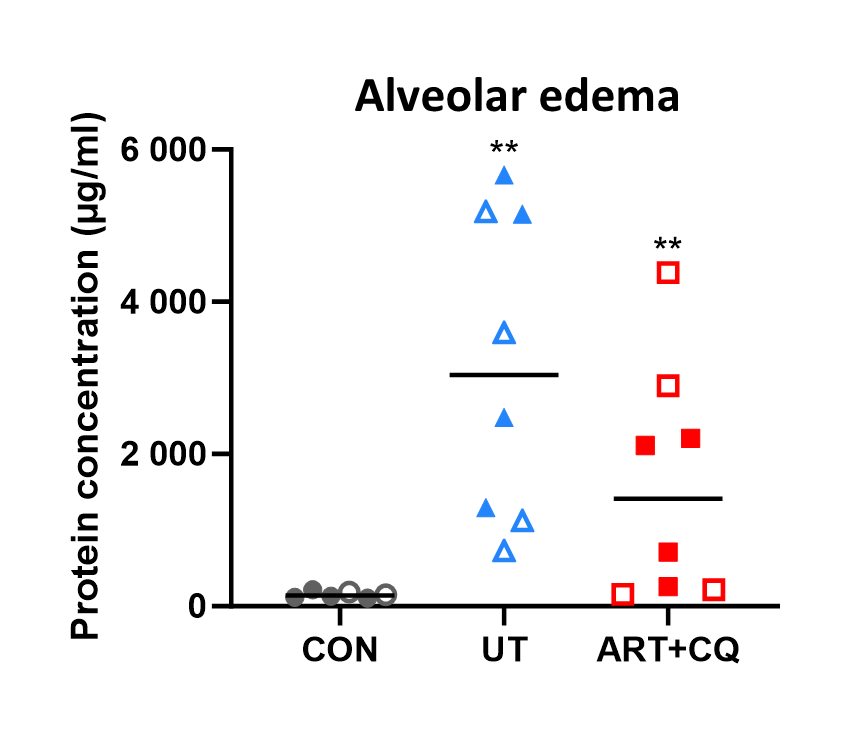

Supplement: S3 Fig — PbNK65-infected C57BL/6 mice were treated daily from 8 until 12 dpi with 10 mg/kg artesunate + 30 mg/kg chloroquine (ART+CQ). Level of alveolar edema was determined by measuring the protein concentration in the BALF. Four mice (full symbols) per condition were selected from the experiment to perform scRNAseq. (TIF) [file ppat.1011929.s003.tif]

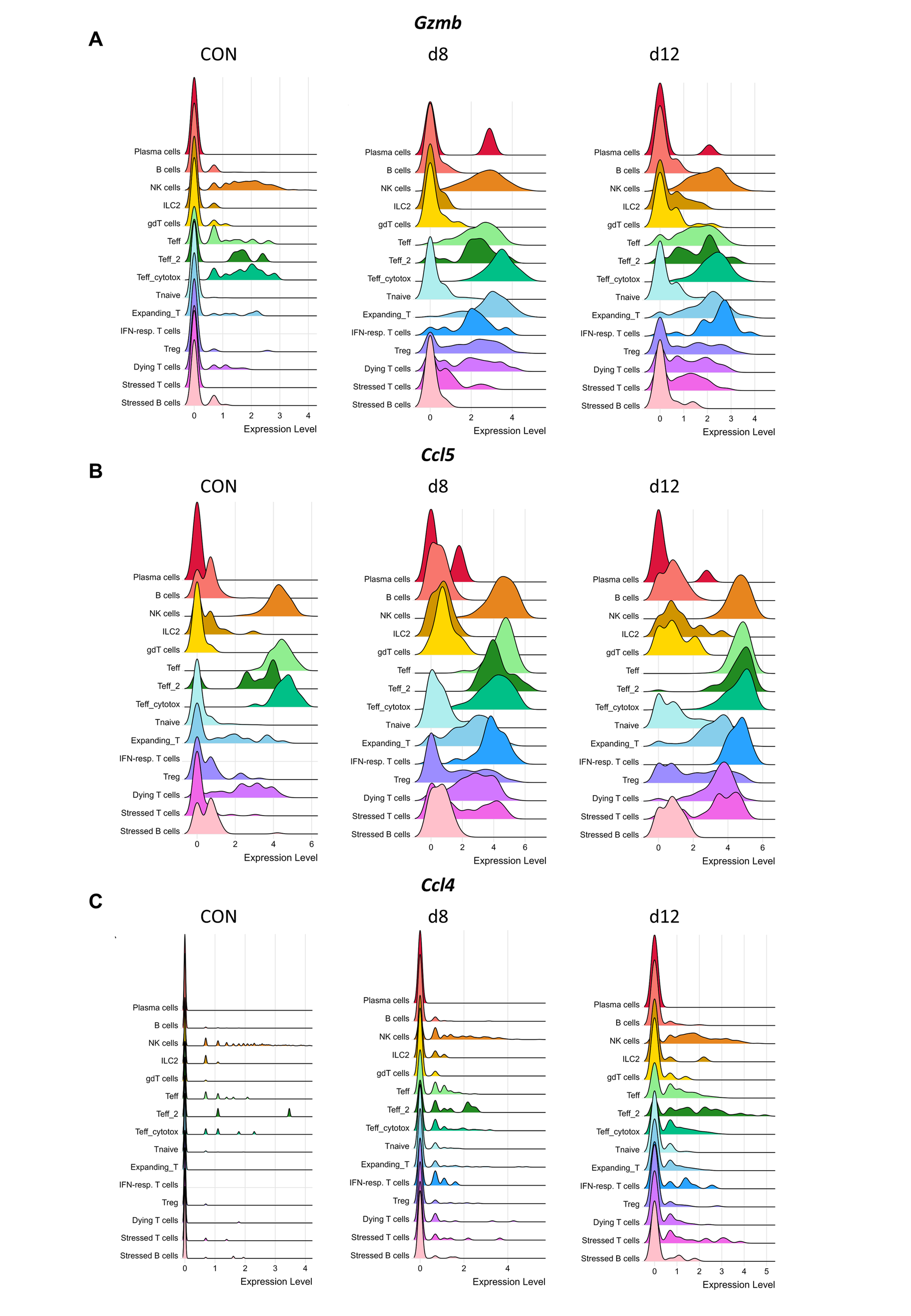

Supplement: S4 Fig — Lymphoid cell clusters (Fig 3) from control (CON), PbNK65-infected (d8) and ART+CQ-treated (d12) mice were checked for the expression of different genes. Expression levels of Granzyme B (GzmB; A), CC chemokine ligand 5 (Ccl5; B) and CC chemokine ligand 4 (Ccl4; C) in the different lymphoid cell populations in all three conditions are shown. (TIF) [file ppat.1011929.s004.tif]

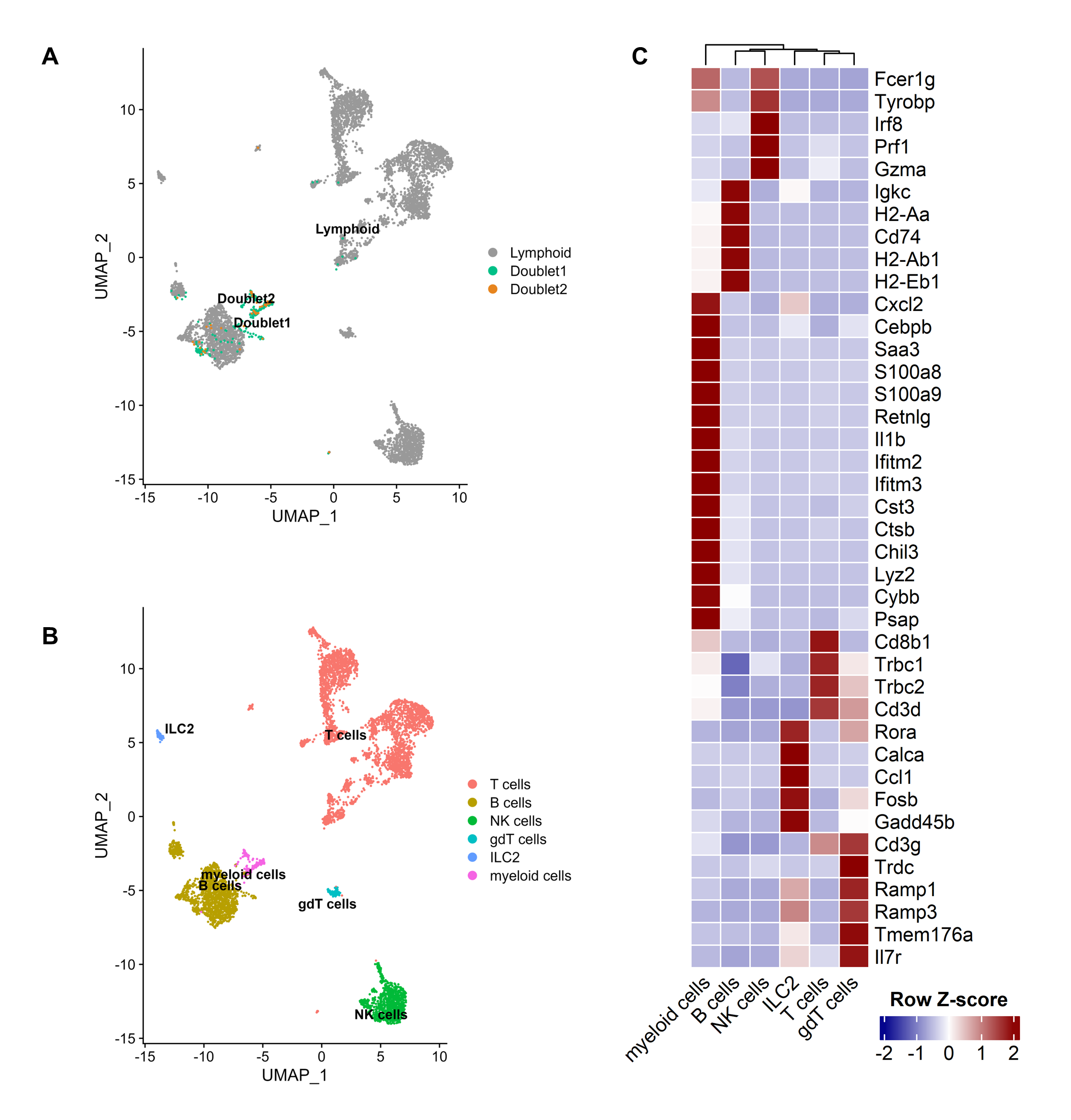

Supplement: S5 Fig — Doublet1 and Doublet2 cluster were identified in the myeloid analysis (Fig 4) with T cell or B cell markers as marker genes respectively. Therefore, these clusters were added to the lymphoid cells (Fig 3) and clustered again. (A) UMAP plot showing where the Doublet1 and Doublet2 clusters are located. (B) UMAP plot according to the major cell types with Doublet1 and Doublet2 clustering together as a myeloid cell cluster. (C) Heatmap displaying the top 5 markers of each cell type and top 15 of the myeloid cluster (from B) and its expression across all cell types. (TIF) [file ppat.1011929.s005.tif]

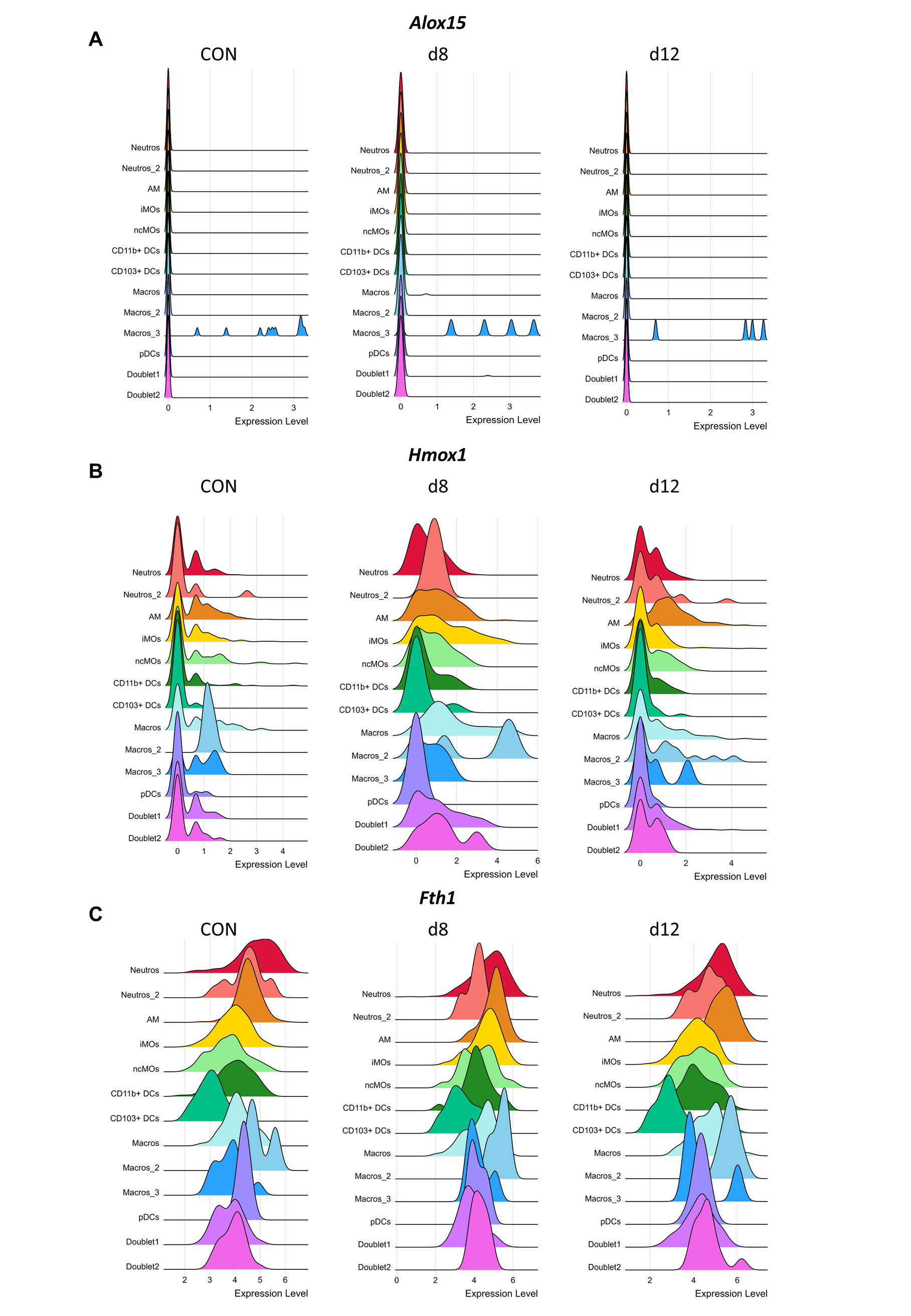

Supplement: S6 Fig — Myeloid cell clusters (Fig 4) from control (CON), PbNK65-infected (d8) and ART+CQ-treated (d12) mice were checked for the expression of different genes. Expression levels of 15-lipoxygenase (Alox15; A), Heme oxygenase 1 (Hmox1; B) and Ferritin heavy chain 1 (Fth1; C) in the different myeloid cell populations in all three conditions are shown. (TIF) [file ppat.1011929.s006.tif]

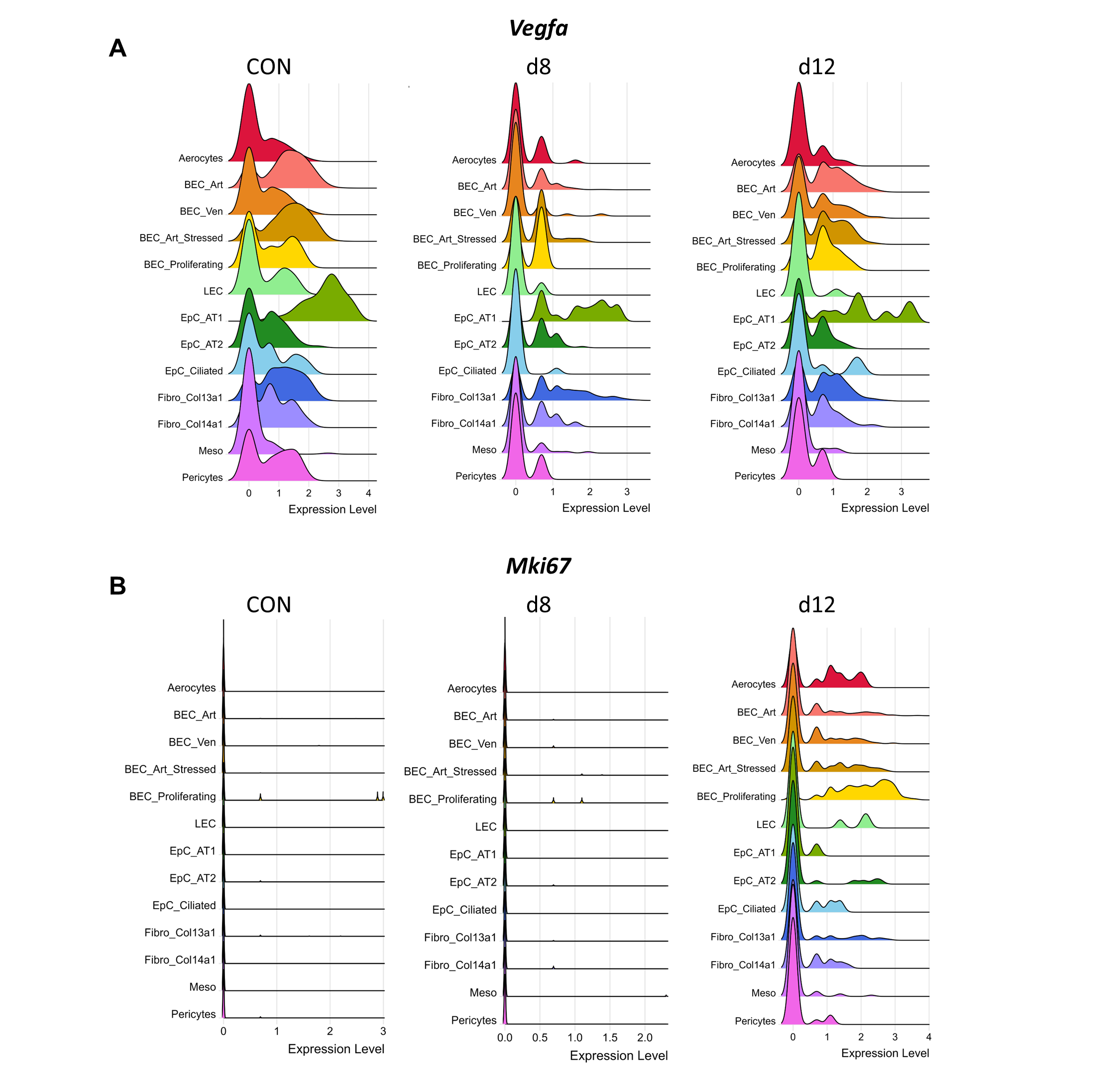

Supplement: S7 Fig — Non-immune cell clusters (Fig 5) from control (CON), PbNK65-infected (d8) and ART+CQ-treated (d12) mice were checked for the expression of different genes. Expression levels of vascular endothelial growth factor (Vegfa; A) and Marker of proliferation Ki-67 (Mki67; B) in the different nonimmune cell populations in all three conditions are shown. (TIF) [file ppat.1011929.s007.tif]

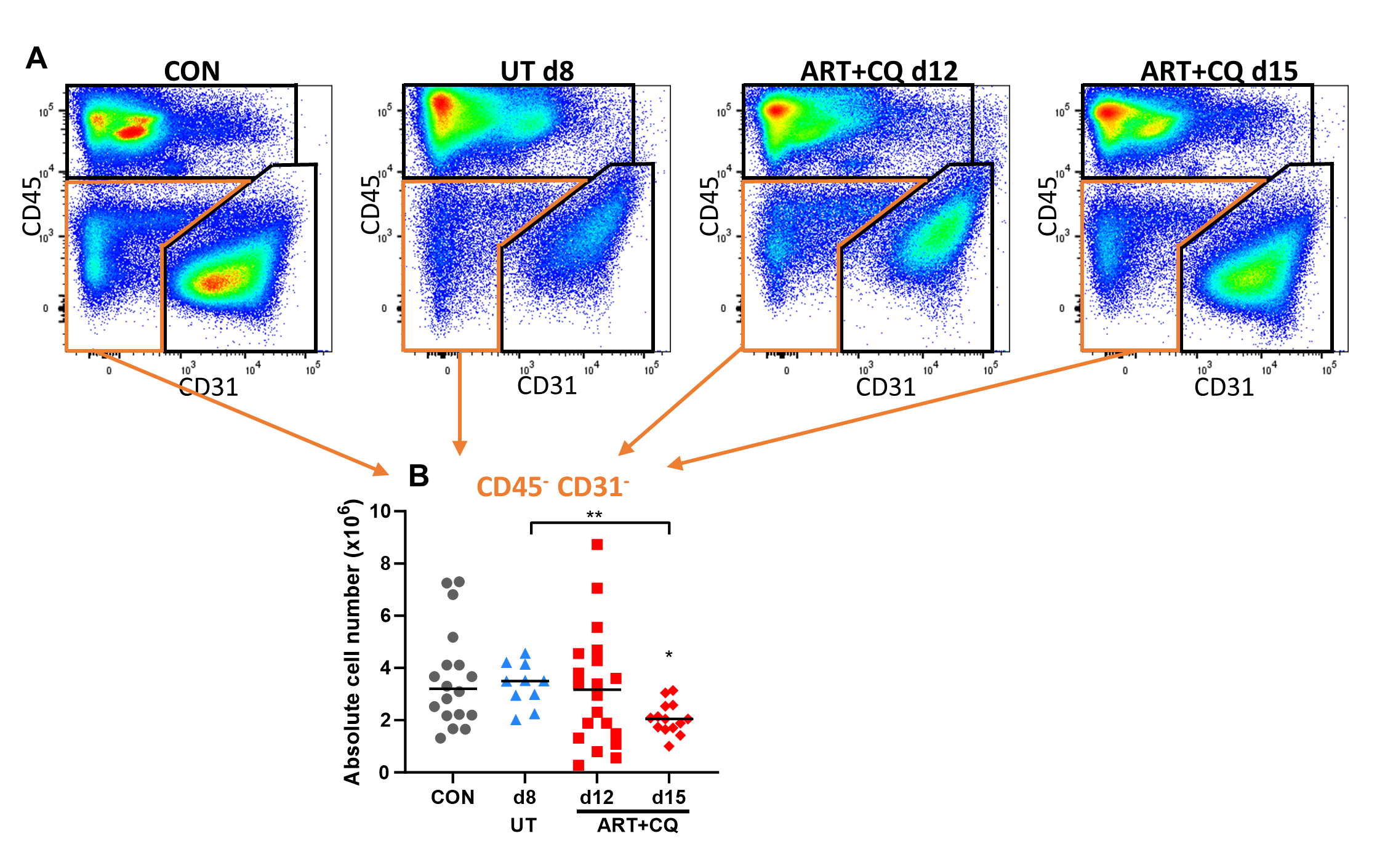

Supplement: S8 Fig — PbNK65-infected C57BL/6 mice were treated daily from 8 until 12 dpi with 10 mg/kg artesunate + 30 mg/kg chloroquine (ART+CQ). Mice were dissected at 8 dpi for the untreated (UT), PbNK65-infected mice and at 12 or 15 dpi for the ART+CQ-treated, PbNK65-infected C57BL/6 mice. Uninfected mice were used as controls (CON). Cells were isolated from the lungs and flow cytometry was performed. (A) Representative FACS plots showing CD45 and CD31 expression on all live single cells, with ECs (CD45- CD31+) and leukocytes (CD45+ CD31-) gated in black and CD45- CD31- population in orange. (B) The absolute number of CD45- CD31+ in the lungs was calculated. Data from two to five experiments. Each symbol represents an individual mouse. Horizontal black lines indicate the median. n = 16–18 for CON, n = 10 for UT d8, n = 20 for ART+CQ d12, n = 14 for ART+CQ d15. (TIF) [file ppat.1011929.s008.tif]

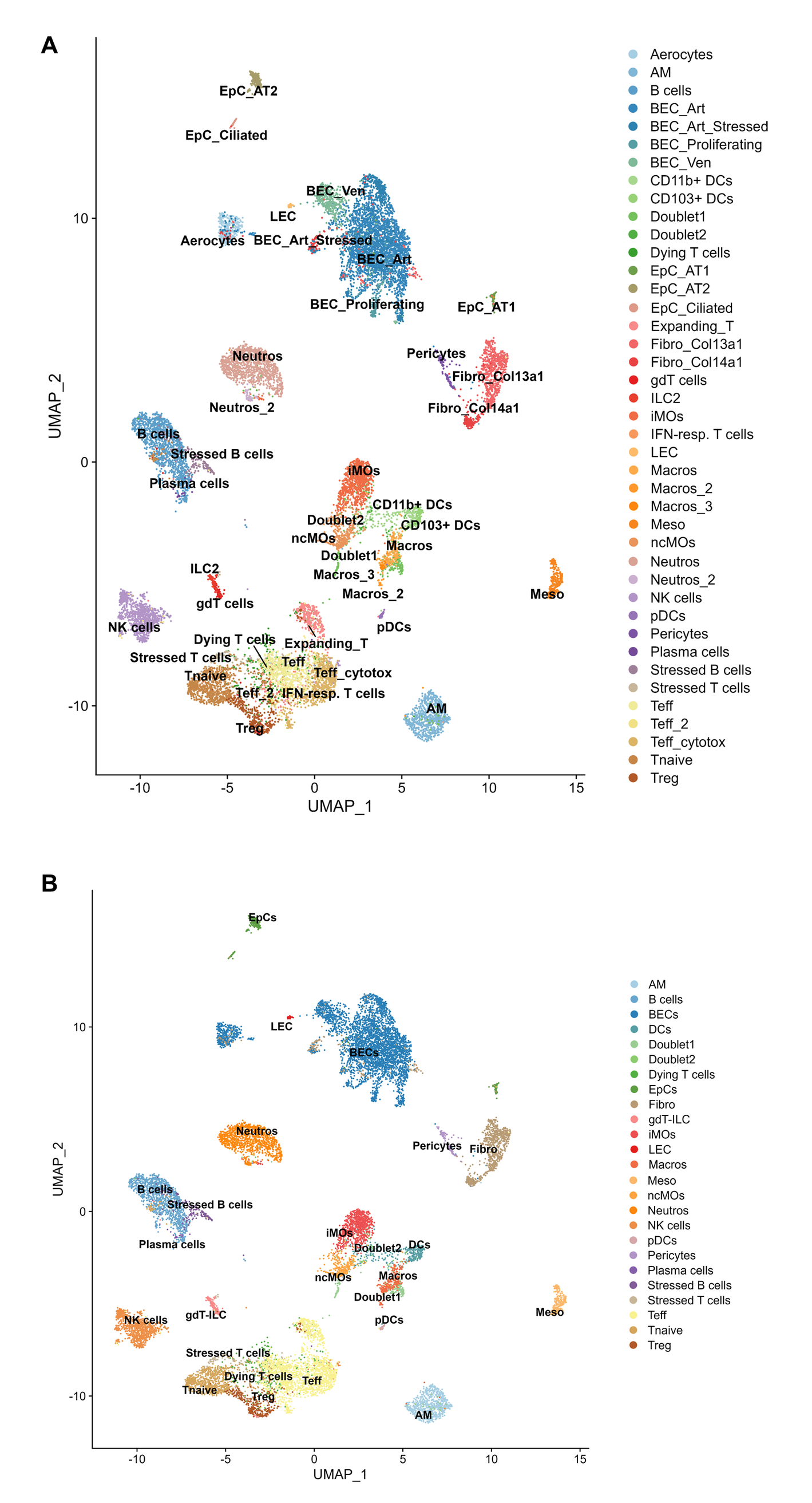

Supplement: S9 Fig — (A) Subcluster identification obtained during the subclustering of lymphoid, myeloid and nonimmune cells, was projected onto the UMAP plot of the original analysis (Fig 2A). (B) Some subclusters were combined into bigger populations for the MultiNicheNet analysis to make the results more robust. UMAP plot of the original analysis showing the clusters used in the MultiNicheNet analysis. (TIF) [file ppat.1011929.s009.tif]
