# Supplementary material for: Single cell RNA sequencing reveals endothelial cell killing and resolution pathways in experimental malaria-associated acute respiratory distress syndrome
Source: PLoS Pathog. 2024 Jan 18;20(1):e1011929. doi: 10.1371/journal.ppat.1011929 (PMC10826972; doi:10.1371/journal.ppat.1011929)
Supplement: S1 Table — Table listing an overview of the antibodies used for flow cytometry including the antigen, fluorophore and the company. 200 000 live single cells were read for each sample. (DOCX) [file ppat.1011929.s010.docx]

**Supplementary Table 1. Antibodies used for flow cytometry**

| **Antigen** | **Fluorophore** | **Company** |
| --- | --- | --- |
| **Endothelial cell panel (200 000 cells read)** | | |
| CD45 | FITC | Biolegend |
| CD40 | PerCP-eFluor710 | eBioscience |
| BrdU | PE | BD Biosciences |
| CD36 | PE-Cy7 | Biolegend |
| ICAM-1 | APC | BD Biosciences |
| CD31 | APC-R700 | BD Biosciences |
| Rae-1 | BV421 | BD Biosciences |
| VCAM-1 | BV650 | BD Biosciences |
| MHCI | BUV395 | BD Biosciences |
| Live/dead | Zombie Aqua™ Fixable Viability Kit | Biolegend |
